# Thermal Activation and Deactivation of Ni‐Doped Ceria Catalysts in CO_2_ Methanation

**DOI:** 10.1002/smsc.202400540

**Published:** 2025-02-06

**Authors:** Mathias Barreau, Davide Salusso, Jinming Zhang, Michael Haevecker, Detre Teschner, Anna Efimenko, Elisa Borfecchia, Kamil Sobczak, Spyridon Zafeiratos

**Affiliations:** ^1^ Institut de Chimie et Procédés pour l’Energie, l’Environnement et la Santé (ICPEES), ECPM UMR 7515 CNRS – Université de Strasbourg 25 rue Becquerel 67087 Strasbourg Cedex 02 France; ^2^ European Synchrotron Radiation Facility CS 40220 Cedex 9 F‐38043 Grenoble France; ^3^ Max‐Planck‐Institut für Chemische Energiekonversion (MPI‐CEC) Stiftstrasse 34‐36 D‐45470 Mülheim a.d. Ruhr Germany; ^4^ Fritz‐Haber‐Institut der Max‐Planck‐Gesellschaft Faradayweg 4‐6 D‐14195 Berlin Germany; ^5^ Interface Design Helmholtz‐Zentrum Berlin für Materialien und Energie GmbH (HZB) Albert‐Einstein‐Str. 15 12489 Berlin Germany; ^6^ Energy Materials In‐situ Laboratory Berlin (EMIL) Helmholtz‐Zentrum Berlin für Materialien und Energie GmbH (HZB) Albert‐Einstein‐Str. 15 12489 Berlin Germany; ^7^ Department of Chemistry INSTM Reference Center and NIS Centers University of Torino 10125 Torino Italy; ^8^ Faculty of Chemistry Biological and Chemical Research Centre University of Warsaw Zwirki, Wigury 101 02‐089 Warsaw Poland; ^9^ Present address: Laboratoire Catalyse et Spectrochimie Université de Caen Normandie, ENSICAEN, CNRS 14000 Caen France

**Keywords:** CO_2_ methanation, in situ and operando spectroscopy, nickel-ceria catalyst, surface exsolution, thermal activation

## Abstract

Discovered almost 130 years ago by P. Sabatier, CO_2_ hydrogenation to methane (CO_2_ methanation) is presently attracting attention as one of the most promising methods for storing intermittent renewable energy in the form of chemical fuels. Ni particles supported by CeO_2_ constitute a very effective, reliable, and reasonably priced catalyst for CO_2_ methanation. Recently a new type of CO_2_ methanation catalyst, consisting of cerium oxide (ceria) nanoparticles doped with nickel (NiCeO_*x*_) in a specific square‐planar configuration with an extremely high‐Ni mass‐specific activity and almost 100% CH_4_ selectivity, was reported. Here, a 50% enhancement in the CO_2_ conversion of the NiCeO_*x*_ catalyst by carefully adjusting the calcination temperature is demonstrated. Notably, thermal aging at 600 °C enhances methanation performance by partially exsolving Ni to the surface, while higher temperatures (750 °C) lead to larger Ni particles, increased CO production, and surface carbon deposition. Several in situ and operando characterization methods are employed to correlate the thermal activation and deactivation of the catalyst with its nanoscale characteristics. Apart from their clear implications for the design of next‐generation Ni‐based CO_2_ methanation catalysts, these findings significantly enhance understanding of the complex interplay and nature of various surface sites involved in CO_2_ hydrogenation.

## Graphical abstract

This work investigates the influence of calcination temperature on the morphology, chemistry, and CO_2_ methanation activity of Ni‐doped ceria catalysts. Optimal calcination temperatures promote the exsolution of small Ni nanoparticles, leading to enhanced methanation activity by creating additional catalytically active sites.

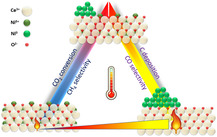

## 1 Introduction

The rapid increase in atmospheric CO_2_ concentrations has led to significant environmental challenges, including global warming and climate change.^[^ ^1^, ^2^ ^]^ Two main strategies have been put forward in order to mitigate CO_2_ emissions while sustaining economic and social development. The first is carbon capture and storage technology, which involves capturing CO_2_ from gases emitted by industry, and storing it by methods such as mineralization or injection into deep underground formations.^[^ ^3^ ^]^ The second is the carbon capture and utilization (CCU) approach which involves the conversion of waste CO_2_ emissions into value‐added products, such as chemicals and fuels.^[^ ^4^, ^5^ ^]^ While the storage of CO_2_ has the potential risk of CO_2_ leakage back into the atmosphere, the CCU approach offers a more sustainable solution by recycling waste CO_2_ emissions and potentially closing the carbon cycle.^[^ ^6^ ^]^ Among the possible reactions to convert CO_2_ to value‐added chemicals, the catalytic hydrogenation of CO_2_ by H_2_ into methane, also called Sabatier reaction, is widely regarded as one of the most practical and appealing solutions.^[^ ^7^ ^]^ This approach is particularly advantageous because the H_2_ needed for the reaction can be produced through water electrolysis using excess wind and solar power generated during periods of low‐energy demand. Accordingly, CO_2_ conversion via the Sabatier reaction allows addressing both the challenges of CO_2_ capture and the storage of intermittent renewable energy, ultimately contributing to net CO_2_ removal from the atmosphere.

Catalysts for the Sabatier reaction typically consist of metal particles dispersed on a high‐surface‐area oxide support.^[^ ^8^ ^]^ Among the various combinations, Ni‐CeO_2_ catalysts stand out for their high CH_4_ yields and lower cost compared to noble metals.^[^ ^9^, ^10^, ^11^ ^]^ While the debate regarding the structure sensitivity of CO_2_ hydrogenation on Ni catalysts remains ongoing,^[^ ^12^, ^13^, ^14^, ^15^ ^]^ recent evidence increasingly indicates that Ni particle size, at least for particles 2 nm and larger, does not significantly impact the overall CO_2_ conversion.^[^ ^16^, ^17^ ^]^ Nonetheless, particle size does influence product selectivity, with larger particles showing a clear trend toward higher CH_4_ selectivity.^[^ ^16^, ^17^, ^18^ ^]^ Regarding the role of the ceria carrier, it is clear that it does not only provide the necessary surface area for Ni but also directly modulates the reactivity through metal–support interactions (MSI) and its capacity to form oxygen vacancies ($V_{O}^{\cdot \cdot }$).^[^ ^14^, ^19^, ^20^, ^21^, ^22^, ^23^, ^24^ ^]^

Despite the fact that the roles of Ni and CeO_2_ may vary depending on the specific catalyst, it is widely accepted that H_2_ dissociates on metallic Ni, whereas CO_2_ primarily adsorbs at $V_{O}^{\cdot \cdot }$ on CeO_2_.^[^ ^9^, ^10^ ^]^ The methanation reaction occurs at the interface between Ni and $V_{O}^{\cdot \cdot }$.^[^ ^25^ ^]^ Consequently, higher dispersion of Ni on ceria increases the number of interfacial Ni–$V_{O}^{\cdot \cdot }$ sites, thereby enhancing the catalyst's activity.^[^ ^26^ ^]^ Recently our group reported that, in addition the metallic Ni, ionic Ni^δ+^ (2 < δ + < 3) embedded within ceria lattice is highly active for CO_2_ methanation.^[^ ^27^ ^]^ The exceptional Ni mass‐specific activity of ionic Ni is attributed to the unique synergy of Ni–Ce pairs, which effectively activates both H_2_ and CO_2_.^[^ ^27^ ^]^ This was demonstrated using Ni‐doped ceria catalysts, which result in the near‐atomic dispersion of nickel on ceria surface and align well with the goal of optimizing nickel dispersion on the support.

The CO_2_ hydrogenation can proceed through two primary reaction pathways, commonly referred to as the CO and the *formate mechanisms*. In the formate mechanism, CO_2_ adsorbed at oxygen vacancy sites reacts with surface hydrogen to form formate intermediates, that are subsequently reduced to CH_4_. Alternatively, in the CO mechanism, CO_2_ is first reduced to CO via the reverse water–gas shift reaction, followed by CO hydrogenation to CH_4_.^[^ ^28^, ^29^ ^]^

The strength of the MSI, specifically the interaction between Ni and ceria, is a critical factor controlling the reactivity. Weak interactions result in Ni agglomeration, reducing the availability of nickel sites for H_2_ dissociation. Conversely, excessively strong interactions can lead to the spillover of reduced CeO*_x_* species and the encapsulation of Ni particles, which suppresses H_2_ dissociation sites and blocks further hydrogenation reactions.^[^ ^26^ ^]^ Thus, only by finely tuning the MSI effect, one can prevent both Ni particle aggregation and CeO_*x*_ spillover, thereby maintaining a maximum number of Ni–$V_{O}^{\cdot \cdot }$ sites.^[^ ^30^ ^]^

Various approaches can be utilized to modulate MSI effects, including controlling the morphology of the support, the size of the metal particles, or the pretreatment conditions.^[^ ^31^ ^]^ Among these, adjusting the catalyst's pretreatment is the most straightforward method. MSI effects are typically observed with reducible oxide supports, making the control of temperature or the choice of reducing agents during treatment a key strategy.^[^ ^30^, ^32^ ^]^ Recent studies, however, have highlighted that the oxidation of the catalyst prior to the reaction—commonly referred to as the calcination step—can also play a decisive role in determining catalytic performance.^[^ ^26^, ^33^, ^34^, ^35^ ^]^

Overall, it can be argued that extensive research on Ni/CeO_2_ has established general guidelines for the rational design of improved CO_2_ methanation catalysts. However, finding a single material that encompasses all these characteristics remains challenging. First, it is hard to synthesize catalysts with atomically dispersed nickel using standard metal precursors, while is even more challenging to avoid sintering and maintain a maximum dispersion on the support under reaction conditions.^[^ ^36^ ^]^ Furthermore, Ce^4+^ reduction is kinetically slow over pure CeO_2_ materials and generally requires severe temperature conditions to favor it. The reducibility and the formation of oxygen vacancies on ceria is enhanced either by modification of its physicostructural properties (size, morphology)^[^ ^37^, ^38^ ^]^ or through the incorporation of metal dopants into its lattice.^[^ ^39^, ^40^ ^]^

Our recent research demonstrated that Ni‐doped ceria nanoparticles catalysts combine both excellent Ni dispersion and enchanced ceria reducibility, resulting in exceptional Ni mass‐specific activity.^[^ ^27^ ^]^ In the present article, we examine how the catalyst can be thermally activated and deactivated upon oxidation pretreatments and how these processes are related to changes in its chemical state. Catalytic tests show that treating the Ni‐doped CeO_2_ nanoparticles catalyst at moderate temperature (600 °C) under oxidative conditions leads to improved activity for CO_2_ methanation reaction combined with up to 99% methane selectivity. Application of postmortem, in situ, and operando analytical methods reveals how the combination of Ce^3+^, ionic nickel species, and small Ni particles is particularly active for CO_2_ hydrogenation, contrary to the common view that pure metallic nickel is indispensable for the reaction. Of more significance, it is shown that generation of big Ni particles to the surface under harsh aging conditions (750 °C) causes a decline of the performance with increased CO production and subsequent carbon deposition that may affect the long‐term stability of the material.

## 2 Results and Discussion

### 2.1 Effect of the Calcination Temperature on NiCeO_*x*_ Characteristics

#### 2.1.1 In Situ XRD During Calcination in Air

**Figure 1:**
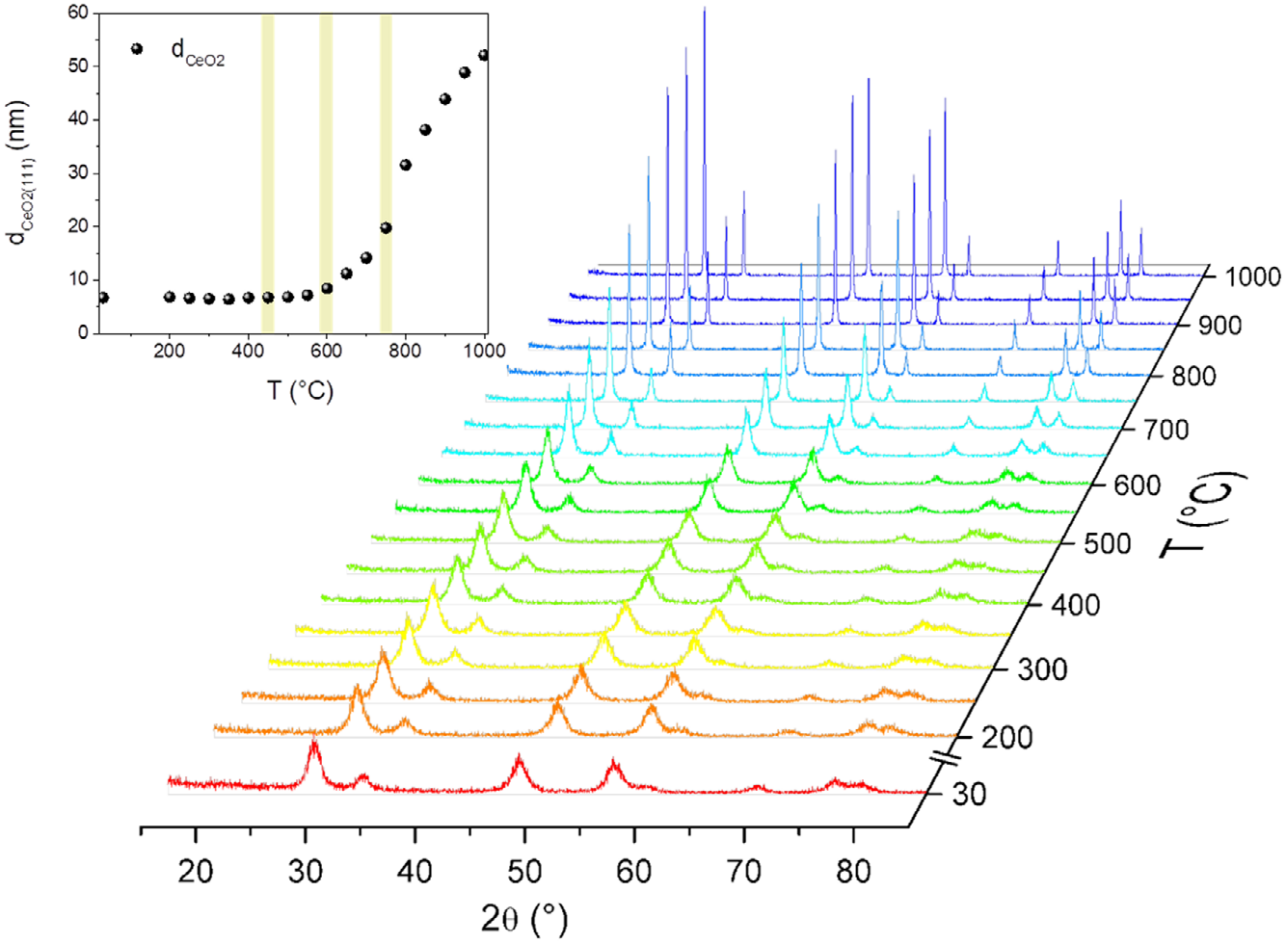
In situ XRD patterns measured between 25 and 1000 °C during annealing in synthetic air flow. The upper‐left graph illustrates the variation of d_CeO2(111)_, reflecting the increase in ceria crystallite size, calculated from the XRD peaks as a function of temperature.

In situ temperature‐programmed X‐ray diffraction (XRD) measurements in synthetic air flow were performed to get insights of the thermally driven modification of the NiCeO_*x*_ crystal structure. The XRD profiles shown in **Figure** **1** and Figure S1 (Supporting Information) correspond to the ceria cubic fluorite phase (JCPDS 34‐0394) which remains stable up to 1000 °C. On the other hand, Ni or NiO diffraction peaks are not detected suggesting high dispersion of Ni species (the nickel content of NiCeO_*x*_, measured by ICP–OES, is 3.6 at%), in accordance with previous results on Ni‐doped ceria.^[^ ^41^, ^42^ ^]^ Upon annealing, the diffraction peaks become narrower while their position is slightly shifted implying increase of ceria crystalline size and lattice parameter, respectively. The evolution of the crystallite size (d_CeO2_) as a function of temperature, calculated by the Scherrer equation of the main (111) reflection peak, is included in Figure 1. As shown, up to 600 °C, d_CeO2_ only slightly increases from 6.7 to 8.4 nm. However, above this temperature there is a rapid growth of the crystallite size (i.e., 20 nm at 750 °C) due to Oswald ripening process assisted by the elevated temperature.^[^ ^43^ ^]^ The ceria cubic fluorite lattice parameter (not shown) increases with temperature as well, reflecting expansion of ceria lattice due to thermal expansion phenomena (i.e., the XRD data are recorded with the sample at elevated temperature).^[^ ^44^, ^45^, ^46^ ^]^

Based on the in situ XRD results, NiCeO_*x*_ catalysts calcined at 450, 600, and 750 °C were selected for further investigation. Specifically, the choice of 450 °C as the minimum calcination temperature was to ensure the removal of residual organic compounds related to the synthesis process.^[^ ^47^ ^]^ At 600 °C, a slight increase in d_CeO2_ is observed, indicating the onset of thermally induced transformation in the ceria crystalline structure. Finally, at 750 °C, the d_CeO2_ increases significantly compared to its initial value, indicating a substantial transformation in the catalyst's structure. For the sake of brevity, hereafter we indicate the catalysts calcined at 450, 600, and 750 °C as NiCe‐450, NiCe‐600, and NiCe‐750, respectively. Following H_2_ activation or methanation reaction, samples are denoted by appending symbols “H_2_” or “R” at the end, such as NiCe‐600H_2_ or NiCe‐600R.

#### 2.1.2 Physicochemical Characterization of the Catalysts Reduced in H_2_

**Figure 2:**
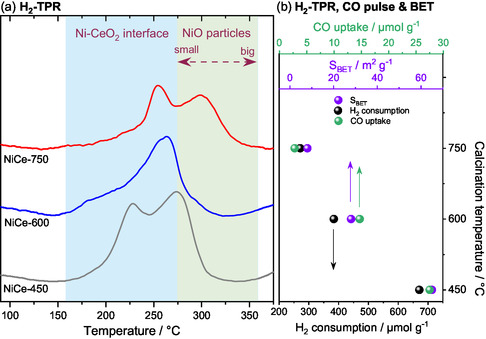
a) TPR and the corresponding H_2_ consumption of NiCeO_*x*_ catalysts calcined at three temperatures. The peaks are normalized to the same height for facile comparison of the peak profiles. b) The H_2_ consumption, BET surface area and CO uptake of the catalysts as calculated by H_2_‐TPR, N_2_‐BET and CO pulse chemisorption measurements, respectively.

The impact of the calcination temperature on the reducibility of the catalyst was investigated using H_2_ temperature‐programmed reduction (H_2_‐TPR) analysis. **Figure** **2**a displays the H_2_‐TPR profiles of the catalysts calcined at the three characteristic temperatures. It's important to note that the maximum reduction temperature in the H_2_‐TPR experiments was 400 °C which corresponds to the activation temperature used in subsequent CO_2_ methanation reaction tests. Consequently, the H_2_‐TPR profiles primarily reflect surface reduction, as bulk ceria typically reduces at around 850 °C.^[^ ^47^, ^48^ ^]^ Based on prior research^[^ ^29^, ^41^, ^47^, ^49^ ^]^ the H_2_‐TPR peaks observed in the NiCe‐450 and NiCe‐600 samples are mainly ascribed to the reduction of adsorbed oxygen species, such as hydroxyl groups and Ni–CeO_2_ interface sites, with potential contribution of highly dispersed NiO particles at temperatures above 270 °C. Notably, the reduction of NiO nanoparticles shifts to higher temperatures with increasing particle size.^[^ ^29^ ^]^ In case of NiCe‐750, the low‐temperature peak is suppressed, and a new intense peak appears around 310 °C, which has been previously associated with the reduction of large NiO particles.^[^ ^29^ ^]^

The BET specific surface area of the calcined catalysts is shown in Figure 2b. As expected, a higher NiCeO_*x*_ calcination temperatures results in lower BET surface areas, indicating sintering and agglomeration of the catalyst particles. This observation aligns with the observed increase in crystallite size found in the XRD results presented above. However, the reactivity of a catalyst is governed by a complex interplay of factors, primarily the density and reactivity of the surface sites.^[^ ^26^ ^]^ A lower surface area does not necessarily correspond to a lower population of active sites. While it is challenging to isolate the effect of surface area from other factors—such as the density and size of nickel particles or the reducibility of ceria—the catalytic results presented below suggest that surface area alone does not provide a straightforward explanation for the observed trends in reactivity.

Furthermore, the overall H_2_ consumption, shown in the same figure, decreases with increasing NiCeO_*x*_ calcination temperature, illustrating a linear correlation between H_2_ consumption and BET surface area. Since the H_2_‐TPR profiles in our case primarily indicate surface reduction, as previously explained, the reduced H_2_ consumption likely reflects variations in surface area among the catalysts, rather than differences in their reducibility. Furthermore, the H_2_ uptake values shown in Figure 2b are significantly higher than the theoretical value for the complete reduction of Ni^2+^ (estimated around 227 μmol g^−1^) confirming the contribution of ceria to the reduction profiles.

The CO pulse chemisorption results of the reduced samples, included in Figure 2b, align with the observed decrease in H_2_ consumption and BET surface areas with increasing calcination temperature. Although CO pulse chemisorption is known to be challenging for estimating Ni dispersion on ceria‐containing catalysts,^[^ ^26^, ^50^ ^]^ the data show that the NiCe‐450 sample exhibits the highest CO uptake, which gradually decreases with the calcination temperature. This indicates a gradual decline in the ability of NiCeO_*x*_ to diffuse and store chemical species that could be necessary for CO_2_ conversion.

#### 2.1.3 In Situ AP‐XPS, AP‐HAXPES, and NEXAFS Measurements in O_2_ and H_2_ Atmospheres

**Figure 3:**
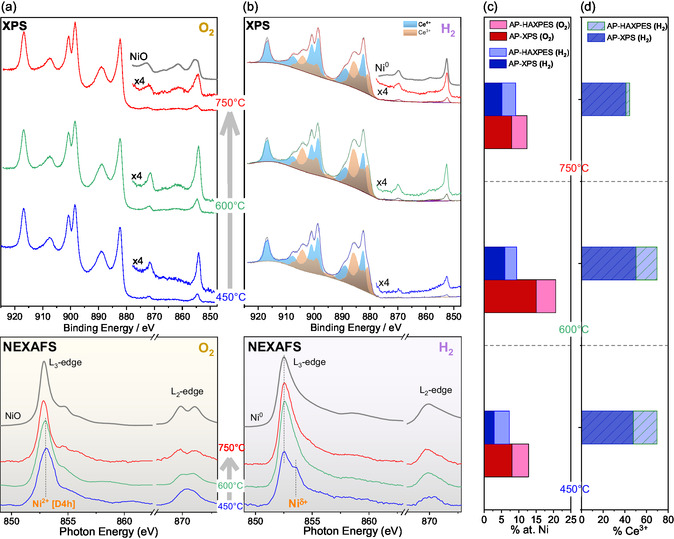
Ce 3d and Ni 2p AP‐XPS (*hv *= 1065 eV) and Ni L_3,2_‐edges NEXAFS spectra of a) NiCe‐450, NiCe‐600 and NiCe‐750 measured at 400 °C in 1 mbar O_2_ and b) NiCe‐450H_2_, NiCe‐600H_2_, and NiCe‐750H_2_ measured at 400 °C in 1 mbar H_2_. The Ce 3d peak is deconvoluted into Ce^4+^ (light blue) and Ce^3+^ (light orange) components while the Ni 2p_3/2_ is magnified by 4 to better distinguish the spectral features. At the top of each figure, the Ni 2p spectra measured in reference NiO and Ni^0^ bulk samples are included for comparison. c) The nickel atomic concentration (in %) calculated based on the Ni 2p and Ce 3d peak areas. d) The portion of Ce^3+^ in the overall Ce 3d spectrum in 1 mbar H_2_ according to the peak fitting presented in Figure 3b. Results from both AP‐XPS and AP‐HAXPES measurements are included in (c) and (d) to emphasize the depth distribution.

Near‐ambient pressure soft and hard XPS (AP‐XPS and ambient pressure‐hard X‐ray photoelectron spectroscopy (AP‐HAXPES) respectively) coupled with NAP‐AP‐NEXAFS spectroscopies were employed to probe the surface state of NiCeO_*x*_ under oxidative and reducing conditions. **Figure** **3**a shows the Ce 3d and Ni 2p spectra regions measured under 1 mbar O_2_ at 450, 600, and 750 °C. Within the Ce 3d spectra, six distinct peak features are observed, notably including an intense peak at 916.8 eV characteristic of Ce^4+^ state. The similarity of the Ce 3d peak to previously published spectra of samples calcined at atmospheric pressure^[^ ^41^ ^]^ suggests complete oxidation of ceria despite the relatively low O_2_ pressure of the AP‐XPS measurement conditions.

The Ce 3d peak shape remains identical across all three calcination temperatures, confirming the maintenance of ceria in Ce^4+^ oxidation state. Conversely, the Ni 2p peak undergoes modifications with the calcination temperature. Specifically, while the BE of the main Ni 2p_3/2_ peak remains constant at 854.4 ± 0.1 eV, the relative intensity of the accompanying satellite feature on the higher BE side increases with the calcination temperature, indicating alterations in the electronic structure of nickel.^[^ ^51^ ^]^ Additionally, the Ni 2p peak shape differs from that of bulk NiO reference spectrum included at the top part of the figure, suggesting a distinct chemical environment around Ni cations compared to bulk nickel oxide (e.g., NiO). Beyond peak shape, the calcination temperature impacts the intensity of the Ni 2p peak, reflecting changes in the nickel surface concentration, as will be discussed below.

After each calcination step, NiCeO_*x*_ catalysts were treated at 400 °C in 1 mbar H_2_ to evaluate the influence of the calcination temperature on the catalyst's reducibility. The Ce 3d spectra, shown in Figure 3b, exhibit significant differences from the previous state in O_2_, manifesting reduction of Ce^4+^ to Ce^3+^. Changes upon annealing in H_2_ are also observed in the Ni 2p peak, which shifts to 852.6 ± 0.1 eV, indicating the reduction of the prior ionic nickel state to metallic Ni^0^.^[^ ^52^ ^]^ However, both the BE position and peak shape differ from that of the reference bulk Ni^0^ spectrum included at the top of Figure 3b, indicating a possible variation in the electronic state of nickel in the reduced NiCe samples compared to bulk metallic Ni^0^.

Recording XPS spectra using 4900 eV photon energy (AP‐HAXPES) allows increasing the analysis depth of the photoemission measurements about 9 times compared to AP‐XPS (from 1.8 to 16.2 nm). As shown in Figure S2 (Supporting Information), the relative intensity of Ni 2p in the AP‐HAXPES spectra differs from that of AP‐XPS, while closer inspection of the Ce 3d spectra in H_2_ indicates that different analysis depths exhibit different ceria reduction degrees. To quantify the differences in the surface state across various treatments, we compared the oxidation state of ceria, estimated by Ce 3d peak fitting, with the nickel atomic ratio calculated using the Ni 2p and Ce 3d peak areas, as shown in Figure 3c,d, respectively. The concentration of Ni (Figure 3c) is consistently higher in AP‐XPS compared to AP‐HAXPES measurements, likely due to surface enrichment of Ni caused by its segregation from the NiCeO_*x*_ lattice toward the surface. Notably, the surface concentration of Ni increases significantly at 600 °C in O_2_, before decreasing again at 750 °C. This trend is maintained under H_2_ conditions and is more pronounced in AP‐XPS measurements than in AP‐HAXPES, suggesting that these changes are confined to the outer 1–2 nm of the nanoparticles without significantly affecting their interior.

The amount of surface nickel is crucial for understanding the influence of calcination temperature on the catalyst's reactivity. Given the analysis depth of AP‐XPS (≈1–2 nm), the %Ni values shown in Figure 3c directly reflect relative changes in the fraction of Ni atoms exposed on the surface after different treatments. By combining %Ni data from AP‐XPS with those from AP‐HAXPES, which has an analysis depth of 16 nm, it is possible to estimate the excess nickel concentration at the surface relative to the bulk—a measure of nickel surface dispersion. As indicated in Table S1 (Supporting Information), the surface‐exposed nickel in the NiCe‐600 sample increases by approximately 2.5 times compared to the NiCe‐450 sample. However, when the calcination temperature is raised to 750 °C, the amount of surface‐exposed nickel decreases, returning to levels similar to those of the NiCe‐450 sample. It is important to note that AP‐XPS alone cannot directly determine whether the observed decrease in nickel concentration for the NiCe‐750 sample results from nickel migration into the ceria bulk or changes in nickel particle morphology, such as agglomeration. However, complementary STEM/EDX results presented below, along with the relatively stable %Ni values obtained from AP‐HAXPES (Table S1, Supporting Information), strongly support the agglomeration scenario.

The calcination temperature also influences ceria reducibility as revealed by the analysis of Ce 3d spectra. Fitting the Ce 3d peak using reference Ce^3+^ and Ce^4+^ spectral lines shows that NiCe‐450H_2_ and NiCe‐600H_2_ have ≈50% Ce^3+^, while NiCe‐750H_2_ exhibits a lower degree of reduction compared to the other two, with around 40% Ce^3+^ (Figure 3d). Furthermore, the Ce^3+^ percentage obtained from AP‐HAXPES (Figure S2, Supporting Information) is systematically lower than that in AP‐XPS, which is a clear evidence that ceria reduction takes place preferentially at the surface of the nanoparticles.

In situ NEXAFS investigation at the Ni L‐edge supplemented the photoemission measurements, providing additional insights into the electronic and geometric structure of nickel. Please note that soft X‐rays were used for the NEXAFS spectra which in the TEY mode has a probing depth in the order of 5–10 nm,^[^ ^53^ ^]^ comparable to that of XPS. The lower panels of Figure 3a,b depict the Ni L_3,2_‐edge spectra collected in O_2_ and H_2_ atmospheres respectively, at varying temperatures. The Ni L_3,2_‐edge of NiCe‐450 is indicative of ionic Ni^δ+^ species (δ^+^ is estimated between +2 and +3) in square planar coordination embedded in ceria lattice, as discussed in detail in our previous work.^[^ ^27^, ^47^ ^]^ Upon calcination at higher temperatures, the centroid of the Ni L_3_‐edge peak gradually shifts toward lower photon energies, accompanied by the appearance of a well‐defined doublet at the Ni L_2_‐edge peak, suggesting gradual formation of Ni^2+^ with typical octahedral symmetry. Although the low spectral signal prevents precise fitting of the Ni L‐edge, the peak shape of NiCe‐600 still suggests a significant proportion of square planar Ni^δ+^. In contrast, the spectrum obtained for the NiCe‐750 sample closely resembles that of the NiO reference, revealing a higher proportion of octahedral Ni^2^^+^ species.

Similarly, differences are observed between the Ni L_3,2_‐edge spectra of the samples in H_2_ atmosphere shown in the lower panel of Figure 3b. In the case of NiCe‐450H_2_, the double‐peak shape of the Ni L_3_‐edge, with features at 852.6 and 853.4 eV, indicates a mixed reduced/oxidized state. This includes Ni^δ^^+^ oxidation state formed due to its interaction with Ce^3^^+^ within the ceria lattice, as detailed previously.^[^ ^27^ ^]^ In contrast, Ni appears predominantly reduced in the NiCe‐600H_2_ and NiCe‐750H_2_ samples, although the Ni L_2_‐edge peak shape still suggests the presence of small amounts of ionic Ni.

### 2.2 Effect of the Calcination Temperature on the CO_2_ Methanation Performance of NiCeO_*x*_

**Figure 4:**
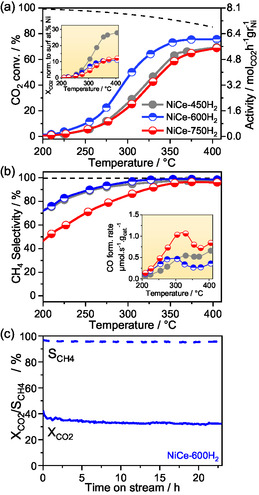
Catalytic performance of NiCe‐600H_2_ and NiCe‐750H_2_ samples. a) CO_2_ conversion (*X*_CO2_) as a function of temperature as well as Ni mass‐based activity (mol_CO2_ h^−1^g_Ni_^−1^), b) CH_4_ selectivity (S_SH4_) as a function of temperature and CO formation rate expressed in μmol_CO_ s^−1^g_cat_^−1^, and c) The *X*_CO2_ and *S*_CH4_ of NiCe‐600H_2_ catalyst at constant temperature of 298 °C as a function of the reaction time. Reaction conditions: CO_2_:H_2_  1:4, GHSV = 12 000 h^−1^, 1 bar.

The CO_2_ conversion (*X*_CO2_) and CH_4_ product selectivity (*S*_CH4_) of NiCeO_*x*_ catalyst calcined at 450, 600, and 750 °C are compared in **Figure** **4**a,b, respectively. Prior to the catalytic tests, all catalysts were activated in 1 bar H_2_ at 400 °C for 30 min. As expected, regardless the calcination pretreatment, both *X*_CO2_ (Figure 4a) and *S*_CH4_ (Figure 4b) increase with temperature and reach a plateau at around 350 °C. The *X*_CO2_ increases considerably after calcination at 600 °C (NiCe‐600H_2_) but declines at higher calcination temperature (NiCe‐750H_2_). A 20% CO_2_ conversion gain is obtained near 300 °C by increasing the calcination temperature from 450 to 600 °C. The maximum *X*_CO2_ (≈76%) is measured at 375 °C for NiCe‐600H_2_. The apparent CO_2_ methanation activation energy (*E*_app_), calculated from the Arrhenius plots (not shown), is around 82 kJ mol^−1^ for all three catalysts, significantly lower compared to previous reported *E*_app_ of Ni‐based catalysts (typically around 120 kJ mol^−1^).^[^ ^54^, ^55^ ^]^ Compared to the catalytic performance of supported Ni/CeO_2_ catalysts reported in the literature, as summarized in the supporting information of reference,^[^ ^27^ ^]^ the NiCe‐600 catalyst demonstrates comparable or even superior activity to catalysts with significantly higher nickel loading.

The conversion normalized by the Ni surface concentration, estimated from the AP‐XPS measurements of Figure 3c, is shown in the inset of Figure 4a. This graph suggests that when considering the surface nickel content, NiCe‐450H_2_ is intrinsically more active than NiCe‐600H_2_. Thus, the higher *X*_CO2_ observed for NiCe‐600H_2_ in Figure 4a results from the increased surface nickel content in this sample due to the preceding calcination step. However, the reaction sites on the surface of NiCe‐450H_2_ are still more efficient for CO_2_ conversion to CH_4_ (see inset of Figure 4a) indicating that this catalyst possesses sites with a higher turnover frequency. This observation is in line with the high intrinsic activity of interstitial ionic Ni^δ+^ species on ceria, as previously explained.^[^ ^27^ ^]^

We turn now our attention to the selectivity to CH_4_ (Figure 4b) which above 300 °C exceeds 95% for both NiCe‐450H_2_ and NiCe‐600H_2_. In contrast, the NiCe‐750H_2_ catalyst has considerably lower selectivity to CH_4_ (i.e., higher CO production) compared to the other two. Further comparison of the CO formation rates shows that this production is doubled at 300 °C as compared to NiCe‐450H_2_ and NiCe‐600H_2_. The significant changes in the surface state of the catalysts with calcination temperature, clearly observed in the H_2_‐TPR and in situ spectroscopic results, are responsible for the observed shifts in selectivity among catalysts calcined at different temperatures, as will be discussed further below.

The stability of NiCe‐600H_2_ catalyst was examined over a period of 23 h at 298 °C. As shown in Figure 4c, apart from a small drop in conversion within the first 30 min, both *X*_CO2_ and *S*_CH4_ remain relatively stable over time, indicating good thermal stability and resistance to carbon deposition.

### 2.3 In Situ XAS of NiCe‐600 and NiCe‐750 Catalysts

Our previous X‐ray absorption near edge structure (XANES) and EXAFS study of NiCe‐450 identified two distinct Ni‐ion types.^[^ ^27^ ^]^ The first type resembles Ni^2+^, but has a coordination environment that deviates from the standard octahedral structure of bulk NiO. These Ni ions were associated with isolated oxidized Ni particles of ≈2 nm in size. The second type consists of atomically dispersed Ni clusters on ceria, likely in an oxidation state higher than +2, possibly corresponding to the Ni^δ+^ species identified by NEXAFS. Notably, the reducibility of these two ionic Ni species in H_2_ is quite different, with the 2 nm Ni particles being more readily reduced compared to the atomically dispersed Ni clusters.^[^ ^27^ ^]^ Building on our previous work,^[^ ^27^ ^]^ we analyze the XANES and FT‐EXAFS spectra of NiCe‐600 and NiCe‐750 catalysts measured under H_2_ activation and reaction conditions.

**Figure 5:**
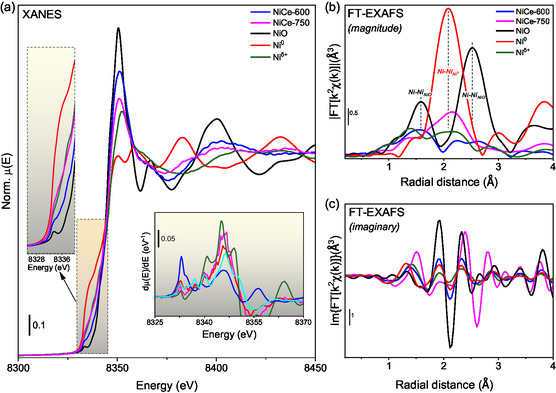
NiCe‐600 (blue line), NiCe‐750 (magenta line), NiO (black line), Ni metallic (red line), and Ni^δ+^ (green line, from another study.^[^ ^27^ ^]^). a) XANES and FT‐EXAFS, b) magnitude, and c) imaginary components. FT‐EXAFS was extracted in the 2.3–10 Å^−1^ range. Details of XANES pre‐edge and its first derivative are reported in panel inset of (a).

**Figure** **5** shows the Ni K‐edge XANES and FT‐EXAFS spectra of the freshly calcined NiCe‐600 and NiCe‐750 catalysts, measured at 1 bar He and room temperature, alongside reference spectra of bulk NiO, metallic Ni foil, and Ni in square planar coordination adapted from another study^[^ ^27^ ^]^ (hereafter referred to as Ni^δ+^). It is important to note here that, although the spectrum of Ni^δ+^ might not precisely represent nickel existing solely in square planar coordination, it is used here as a descriptor for this state since it is currently the only available spectrum that displays nickel with square planar characteristics. Comparison of the Ni K‐edge spectra of NiCe‐600 and NiCe‐750 with reference peaks reveals significant differences in white‐line intensity and the structured shoulder in the rising edge region (clearly visible in the first‐derivative spectra shown in the inset of Figure 5a). These differences suggest that nickel exists in a multiple‐oxidation state distinct from that of the pure NiO and Ni^δ+^ phases.

**Figure 6:**
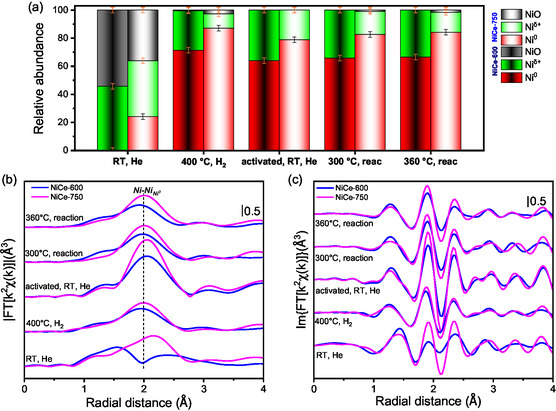
a) NiO, Ni, and Ni^δ+^ relative abundances evaluated through LCF analysis of NiCe‐600 (left columns) and NiCe‐750 (right columns). Representative FT‐EXAFS spectra reported as b) magnitude and c) imaginary components of NiCe‐600 (blue lines) and NiCe‐750 (pink lines) selected from the steady states from protocol represented in Figure S4 (Supporting Information). FT‐EXAFS was extracted in the 2.3–10 Å^−1^ range.

Quantification through LCF using Ni K‐edge spectra of reference Ni^δ+^, NiO, and Ni^0^ samples (**Figure** **6**a) indicates that both NiCe‐600 and NiCe‐750 contain multiple nickel species. Particularly, incorporating the Ni^δ+^ spectrum in the LCF is crucial for accurately fitting the Ni K‐edge of the calcined samples (see also Figure S3, Supporting Information), confirming that a portion of square planar Ni persists after calcination. While the Ni^δ+^ species is preserved also in the NiCe‐750 sample, in this case, part of NiO is replaced by metallic Ni^0^ (Figure 6a). The presence of Ni^0^ for NiCe‐750 is further corroborated by the FT‐EXAFS magnitude and imaginary components (Figure 6b,c), where the Ni–Ni_Ni_ single‐scattering path becomes evident. In contrast, for NiCe‐600, the FT‐EXAFS first and second shells are weak and broad, suggesting a disordered local environment consistent with Ni in square planar coordination. Although metallic Ni^0^ on NiCe‐750 sample was not detected by the photoemission techniques discussed earlier in the manuscript, its potential presence in the core of larger nickel particles cannot be ruled out. Such Ni^0^ may be detectable by Ni K‐edge XAS but remains inaccessible to surface‐sensitive photoemission techniques, which primarily probe the outermost particle regions. In fact, transmission electron microscopy (TEM) measurements presented later support this scenario, revealing the formation of nickel particles up to 120 nm in size in the NiCe‐750 sample.

The samples were subsequently measured in situ according to the protocol outlined in Figure S4 (Supporting Information). After the H_2_ activation step at 400 °C, all Ni^2^^+^ is reduced to Ni^0^ in both samples, while part of Ni^δ+^ is preserved. Notably, the FT‐EXAFS spectra (Figure 6b,c), measured at room temperature after activation, show a more intense Ni–Ni_Ni_ component for NiCe‐750H_2_ compared to NiCe‐600 H_2_. While LCF analysis suggests similar Ni^δ+^/Ni ratios for both catalysts, FT‐EXAFS fitting (Figure S5, Table S2, Supporting Information) indicates a higher Ni–Ni coordination number (CN) for the Ni^0^ fraction in NiCe‐750H_2_, suggesting larger Ni particle sizes.

The catalysts were then exposed to CO_2_:H_2_ (1:4) reaction mixture at RT and heated to 300 and 360 °C. XANES spectra did not present significant variation of Ni^δ+^/Ni ratios (see Figure 6a), indicating stability of both catalysts under reaction conditions. Notably, NiCe‐600R consistently contained a higher proportion of Ni^δ+^ compared to NiCe‐750R. Furthermore, fitting of the FT‐EXAFS collected at RT after reaction revealed that the Ni^0^ fraction in NiCe‐750 retained a higher Ni–Ni CN, indicating that the particle size remained stable throughout the entire experimental protocol. Moreover, the fit revealed that the NiO structure used in the fitting could not fully capture the experimental data (both before and after the reaction), suggesting: 1) a shorter Ni—O bond length compared to NiO (≈1.88 vs. 2.07 Å) and 2) larger Ni—O and Ni—Ni Debye–Waller factors (Table S2, Supporting Information). These findings align with the previously reported complex nature of Ni^δ+^ ions embedded in CeO_2_, which is not adequately captured by the reported FT‐EXAFS fitting.^[^ ^27^ ^]^

### 2.4 Analysis of the Spent NiCe‐600R and NiCe‐750R Catalysts

According to the AP‐XPS results in Section 2.1.3, the improved reactivity of NiCe‐600 compared to NiCe‐450 may stem from an augmentation in surface‐exposed nickel following high‐temperature calcination. However, despite NiCe‐750 possessing comparable surface nickel content to NiCe‐600 and nearly double that of NiCe‐450, it displays lower CO_2_ conversion and CH_4_ selectivity than the other two. This suggests that reactivity is not solely determined by the nickel quantity but also depends on the specific type of nickel sites present, a notion supported by our preceding findings.^[^ ^27^ ^]^ In this section, we look for eventual differences between NiCe‐600R and NiCe‐750R after the catalytic cycle (spent catalysts) that could explain the lower catalytic activity and CH_4_ selectivity of NiCe‐750 despite the fact the two catalyst have similar Ni surface amount (AP‐XPS) and similar electronic states (XANES).

**Figure 7:**
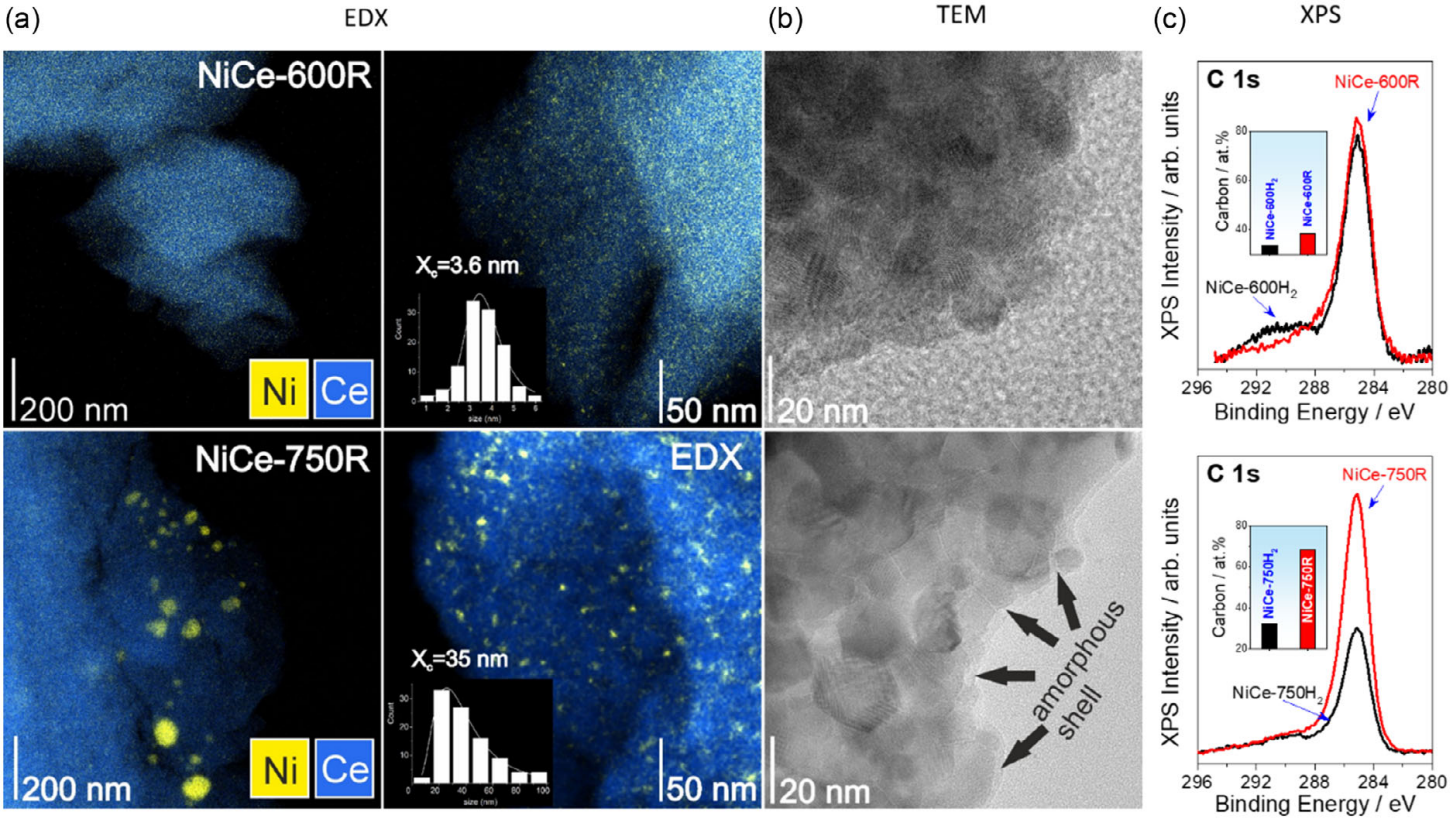
a) EDX elemental mapping and Ni particle‐size distribution histogram (more than 90 particles analyzed) for NiCe‐600R (top) and NiCe‐750R (bottom), b) the corresponding BF‐TEM images, c) C 1s quasi in situ XPS spectra of NiCeO_*x*_ catalysts calcined at 600 and 750 °C recorded just after the H_2_ reduction treatment (black lines) and after CO_2_ hydrogenation (red lines). The bar graphs inserted in the EDX images shows the Ni particles size contribution. Reaction conditions: samples were previously reduced in 7 mbar H_2_ up to 400 °C with 10 °C min^−1^ heating rate followed by 30 min step at 400 °C (NiCe‐600H_2_ and NiCe‐750H_2_). Subsequently, similar heating procedure was applied under 7 mbar of CO_2_:H_2_ reaction mixture with 1:4 molar ratio (NiCe‐600R and NiCe‐750R).^[^ ^66^ ^]^

STEM/EDX mapping images (**Figure** **7**a) of the two catalysts show that NiCe‐600R maintains the small size of Ni particles (<3.5 nm), while NiCe‐750R exhibits significant sintering, leading to the formation of large Ni particles measuring up to 120 nm (see inserted histograms). This observation is consistent with the H_2_‐TPR and XANES results, which indirectly indicate the presence of large nickel particles following aging at 750 °C. However, low‐magnification STEM/EDX mapping images of NiCe‐750R (Figure S6, Supporting Information) show that large Ni particles (>20 nm) are less than 5% of the detected Ni species, and nickel remains highly dispersed over ceria.

Similarly, Raman spectra of NiCe‐600R and NiCe‐750R catalysts (Figure S7, Supporting Information) confirm the continual presence of Ni–O–Ce interfaces and/or oxygen defects induced by Ni, as indicated by bands in the 550–650 cm^−^^1^ range^[^ ^56^, ^57^ ^]^ and the activation of the band at 228 cm^−^^1^, associated with disorder in the CeO_2_ lattice (TO phonon mode).^[^ ^58^ ^]^ However, a notable decrease in the intensity of these bands is observed with increasing aging temperature, in contrast to the intensity of the peak centered at 461 cm^−^^1^, which corresponds to the triply degenerate F_2__g_ mode of the cubic fluorite‐type CeO_2_ structure.

EDX line profiles of NiCe‐750R sample (Figure S8, Supporting Information) show a significant carbon signal at the particle edges, which is not present in the NiCe‐600R sample. This suggests the deposition of a thin carbon layer on the surface of NiCe‐750R, correlating with the potential deterioration of its catalytic performance. Carbon deposition is further confirmed by the TEM images (Figure 7b) showing a thin amorphous layer on the surface of NiCe‐750R, which does not exceed 2 nm in thickness. An equivalent increase in the carbon content of the NiCe‐750R catalyst is also noted in the C 1s XPS spectra recorded after reaction (Figure 7c). The Ce 3d and Ni 2p peak shapes remain practically unchanged in comparison with the spectra of activated catalyst prior the reaction (Figure S9, Supporting Information), in good agreement with the XANES data. However, one can note a significant decrease of Ce/Ni atomic ratio in the case of the NiCe‐750R sample upon reaction (Table S3, Supporting Information), while similar Ce/Ni ratios are obtained for both NiCe‐600H_2_ and NiCe‐600R samples. Carbon over NiCe‐750R sample is therefore preferentially accumulated on ceria rather than on nickel. It is known that CO produced through reverse water–gas shift reaction (CO_2_ + H_2_ → CO + H_2_O) has much higher tendency to dissociate and leave carbon on the surface than CH_4_.^[^ ^59^, ^60^ ^]^ This can explain the higher carbon amount on NiCe‐750R catalyst, which is in line with the higher selectivity of NiCe‐750R toward CO. Overall, analysis of the spent catalysts suggests that NiCe‐600R, which has smaller Ni particle size and higher Ni dispersion, also exhibits improved coke resistance in CO_2_ methanation compared to NiCe‐750R. The significant coke formation observed in the case of NiCe‐750R can be correlated with the higher CO selectivity of this catalyst.^[^ ^66^ ^]^

### 2.5 NiCeO_*x*_ Surface State and CO_2_ Methanation

**Figure 8:**
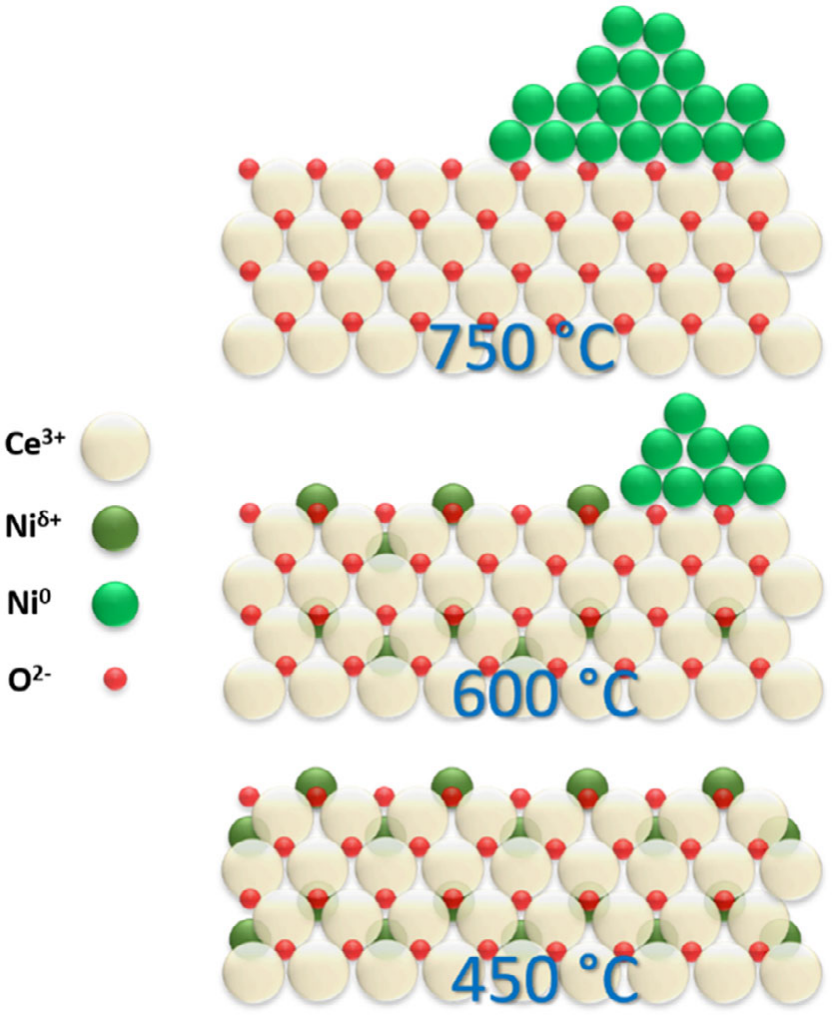
Schematic representation of the proposed surface configuration of NiCeO_*x*_ catalyst formed under H_2_ atmosphere after calcination at the indicated temperatures.

The analysis presented above indicates that the calcination temperature significantly impacts Ni distribution and ceria reducibility, both of which are critical for catalyst performance. Specifically, calcination at 600 °C (NiCe‐600) results in a higher Ni surface concentration compared to NiCe‐450, without significantly compromising Ni dispersion or ceria reducibility. Conversely, higher calcination temperature (NiCe‐750) promotes the formation of large Ni clusters and decreases the concentration of surface Ce^3^^+^ species. Furthermore, there is strong evidence suggesting the coexistence of two nickel oxidation states (metallic Ni^0^ particles and ionic Ni^δ^^+^) within the NiCeO_*x*_ catalyst during CO_2_ methanation. Notably, the proportion of Ni^0^ particles increases at the expense of Ni^δ^^+^ as the calcination temperature rises. As demonstrated by in situ AP‐XPS and NEXAFS data, NiCe‐450 exhibits the highest fraction of Ni^δ^^+^ species and a significant amount of Ce^3^^+^ sites (≈50%) among the analyzed samples. The interfacial Ni^δ^^+^–Ce^3^^+^ sites, schematically illustrated in **Figure** **8**, serve as active centers for CO_2_ methanation, exhibiting significantly higher site‐specific activity compared to conventional Ni^0^ particles–Ce^3^^+^ sites.^[^ ^27^ ^]^

In the NiCe‐600 sample, the surface exsolution of Ni, as confirmed by XPS, XANES, and STEM/EDX analyses, leads to the formation of Ni^0^ particles in contact to Ce^3^^+^ support sites, creating additional active centers for the methanation reaction (Figure 8). The substantial increase in surface Ni content (2.5 times) significantly enhances catalytic activity compared to samples calcined at 450 °C. However, while some Ni^δ^^+^–Ce^3^^+^ interphase sites are preserved at this higher temperature, XANES and NEXAFS analyses indicate that they no longer constitute the majority. As a result, although the increased surface Ni content enhances the overall catalytic performance, the reduction in the number of highly active Ni^δ^^+^–Ce^3^^+^ sites explains why when normalized by Ni surface concentration, the NiCe‐450 sample demonstrates higher activity than NiCe‐600.

High‐temperature calcination at 750 °C affects the ceria crystalline size and leads to nickel agglomeration to bigger particles. These morphological changes result in a reduced concentration of surface Ce^3^^+^ species and, most notably, a decrease in the Ni^δ^^+^–Ce^3^^+^ and Ni^0^ particle–Ce^3^^+^ interfacial sites, explaining the decline in the CO_2_ conversion. The decrease in CH_4_ selectivity, favoring CO production for this catalyst, may arise from two main factors. Lower CH_4_ selectivity of Ni‐CeO_2_ catalysts has been associated with both small Ni particle sizes (<8 nm)^[^ ^16^, ^18^ ^]^ and weaker MSI interactions.^[^ ^24^, ^30^ ^]^ As indicated by STEM/EDX results, the changes in Ni particle size of NiCe‐750 do not align with trends that would enhance the CO production pathway. This leaves the modifications in MSI effects caused by high‐temperature calcination as the most likely explanation for the increased CO production observed with the NiCe‐750 catalyst.

Overall, calcination at a moderate temperature (600 °C) optimally balances the increased Ni surface content with a suitable population of Ni^δ^^+^–Ce^3^^+^ and Ni^0^ particle–Ce^3^^+^ sites. After this treatment the sample achieves high Ni dispersion, while preserving a significant population of interfacial Ce^3^^+^ sites in contact with both metallic Ni^0^ particles and ionic Ni^δ^^+^, resulting in superior CO_2_ methanation activity.

## 3 Conclusion

This work highlights the impact of calcination temperature on the key morphological and chemical characteristics of Ni‐doped ceria catalysts and their correlation with CO_2_ methanation performance. We demonstrate that optimizing the calcination temperature can boost the methanation performance of these catalysts—already exhibiting exceptionally high‐Ni mass‐specific activity due to their unique Ni coordination—by approximately 20%. While increasing the calcination temperature from 450 to 600 °C decreases the number of highly active Ce^3+^–Ni^δ+^ interfacial sites, the exsolution of additional metallic nickel nanoparticles (<4 nm) provides extra active sites that compensate for the loss of Ce^3+^–Ni^δ+^. The highest activity of the sample calcined at 600 °C is thus attributed to the high surface concentration of Ni and the coexistence of both interfacial sites and Ni nanoparticles, which together facilitate the activation of CO_2_ and H_2_. In contrast, further increasing the calcination temperature to 750 °C diminishes the Ce^3+^–Ni^δ+^ sites and leads to the formation of larger Ni particles, ultimately resulting in a decrease in both methanation activity and selectivity. Notably, the increase in CO selectivity accompanying the formation of large nickel particles is followed by significant deposition of amorphous carbon on the catalyst surface, which contributes to the decline in performance. Consequently, gaining a deeper understanding of the complex interplay between Ni and Ce within the solid solution, as well as the influence of heat treatment conditions on the nature of active sites presented in this study, can aid in the design of more effective methanation catalysts with optimized Ni content.

## 4 Experimental Section

4.1

4.1.1

##### Synthesis of Ni‐Doped Ceria Nanoparticles by the Soft‐Template Method

The Ni‐doped ceria nanoparticles (hereafter abbreviated as NiCeO_*x*_ NPs) were synthesized following a synthesis method described in detail elsewhere.^[^ ^47^ ^]^ Briefly, synthesis involved mixing and pyrolysis of Ni and Ce Schiff base metal complexes in oleylamine media. The resulting nanoparticles were obtained by extraction with methanol and a final calcination step at 450 °C to remove oleylamine and form a fine powder. Further calcination at 600 or 750 °C was conducted with 10 °C min^−1^ heating rate for 30 min to obtain NiCe‐600 and NiCe‐750 samples, respectively. The nickel content of NiCeO_*x*_, measured by inductively coupled plasma–optical emission spectrometry (ICP–OES), was 3.6 at% (i.e. 1.3 wt%) and remained identical for every calcined sample since all catalysts came from the same synthesis batch.

##### Catalytic Tests

CO_2_ methanation tests were performed using a U‐shaped fixed‐bed flow reactor with an internal diameter of 6 mm. Three calibrated mass flow controllers (Bronkhorst) were used to introduce the gas mixture at a total flow rate of 12 L h^−1^ g_cata_^−1^, yielding a gas hourly space velocity (GHSV) of ≈12 000 h^−1^ In these tests, the catalyst (100 mg) was mixed with 100 mg of SiC, both sieved to between 150 and 250 μm. Initially, the samples were activated in 1 bar of H_2_ at 400 °C for 30 min. The catalytic tests were then conducted by steps, ranging from 150 to 400 °C, in 1 bar of a CO_2_:H_2_ mixture with a 1:4 molar ratio. The catalytic tests were repeated twice for each sample and the CO_2_ conversion and CH_4_ selectivity difference in each experiment was below 5%.

The outlet gas was analyzed with a gas chromatograph (Agilent 5975C VL MSD) equipped with a Molecular Sieve 5 A column, a PoraPLOT U GC column, and a thermal conductivity detector (TCD) for CO_2_, CH_4_, CO, and H_2_O detection. Due to similar retention times with the carrier gas (He), identifying and measuring H_2_ was not possible.

The CO_2_ conversion $\left(X_{{\text{CO}}_{2}}\right)$ and CH_4_ selectivity $\left(S_{{\text{CH}}_{4}}\right)$ were calculated as follows (Equation (1) and (2)).

(1) $${\text{CO}}_{2}\text{ Conversion}:\;X_{{\text{CO}}_{2}}(\%)=\frac{{\text{CO}}_{2,\text{in}}-{\text{CO}}_{2,\text{out}}}{{\text{CO}}_{2,\text{in}}}\times 100$$

(2) $${\text{CH}}_{4}\text{ Selectivity}:\;S_{{\text{CH}}_{4}}\left(\%\right)=\frac{{\text{CH}}_{4,\text{out}}}{{\text{CH}}_{4,\text{out}}+{\text{CO}}_{\text{out}}}\;\times 100$$

where CO_2out/in_, CH_4,out_, and CO_out_ were the concentrations at inlet/outlet of the reactor. The concentration of each gas was quantified by GC peak area multiplication with the response factor of each gas determined by calibration. Concerning the output concentrations, a carbon balance‐based correction factor was incorporated into the computation to account for flow variance during the test. The apparent activation energy (*E*_a_) was determined from Arrhenius plots in the kinetic regime, where CO_2_ conversion was below 35%.

##### Standard Characterization

N_2_ physisorption measurements were carried out on a Micromeritics Tristar 3000 apparatus. Prior to the N_2_ adsorption–desorption measurements at −196 °C, the calcined samples were degassed at 150 °C for 6 h under primary vacuum. The surface areas were determined according to the Brunauer–Emmett–Teller (BET) method with relative pressures (p/p_0_) ranging between 0.05 and 0.35. Temperature‐programmed reduction experiments (H_2_‐TPR) were carried out in an AutoChem II apparatus (Micromeritics) incorporating TCD. The profiles were obtained after loading 50 mg of sample in a U‐shaped fixed bed reactor and heating up to 400 °C under 20 mL min^−1^ of 10% H_2_/Ar with a 10 °C min^−1^ heating rate. AutoChem II equipment was also utilized to conduct CO pulse chemisorption tests. Prior to the measurements, 50 mg of catalyst were prereduced in 10% H_2_/Ar at 400 °C for 30 min, aligning with the activation temperature used for the catalytic tests. The system was then purged under He at the same temperature and cooled down to 40 °C. CO pulses (10% CO/He) were subsequently introduced until the TCD signal reached a constant value, allowing to obtain the total CO uptake after peak fitting procedure. Raman spectroscopy was performed on a micro‐Raman spectrometer (Horiba LabRam Aramis) with a 532 nm laser as the excitation source. A 10× objective was used to focus the excitation laser, giving ≈2.6 μm‐wide spot while avoiding damaging of the sample with a 1 mW laser power. Elemental analysis was carried out by ICP–OES (Varian 720 ES) after the dissolution of the powdered sample in acidic medium (HNO_3_) followed by filtration of residual particles. The TEM characterization was carried out using a FEI Talos F200X microscope operating at 200 kV. Observations were performed in scanning transmission electron microscopy (STEM) mode using high‐angle‐annular dark‐field imaging. Energy‐dispersive X‐ray spectroscopy (EDX) using a Super‐X system with four silicon drift detectors was applied to the detection of differences in local chemical composition. In situ X‐ray diffraction (XRD) patterns were recorded on a Bruker D8 advance diffractometer operating at 40 kV and 40 mA using Cu Kα radiation (*λ* = 1.5418 Å). The device was equipped with a temperature chamber that gave the possibility to register XRD patterns between room temperature and 1000 °C. XRD patterns were recorded from 20 to 80° at a scan rate of 0.032° s^−1^. The resulting patterns were processed using DIFFRAC.EVA for the crystallite size calculation according to the line broadening of the most intense reflection (i.e., the (111) plan for the fluorite‐type CeO_2_ phase) using the Scherrer equation.

*Quasi‐*in situ X‐ray photoelectron spectroscopic (XPS) measurements were performed using a custom‐build ultrahigh‐vacuum setup precisely described elsewhere.^[^ ^61^ ^]^ Briefly, the setup was composed of reaction and analysis chambers interconnected via a load‐lock chamber allowing fast insertion of the samples into the system. The analysis chamber was equipped with a VSW Class WA hemispherical electron analyzer (150 mm radius) and a monochromatic Al Ka X‐ray source (1486.6 eV) for XPS measurements. The reaction chamber contained a flow‐through reactor equipped with a sample receiving station, a gas manifold at the inlet, and a motorized gas control valve at the outlet connected with a dry scroll pump, allowing for tests in the mbar to the bar range pressure. Prior to the sample insertion, few drops of NiCe nanoparticles dispersed in hexane solution were deposited onto a gold foil and calcined at 600 or 750 °C, following the procedure already reported in “Synthesis of Ni‐Doped Ceria Nanoparticles by the Soft‐Template Method”. Prereduction was conducted in 7 mbar H_2_ at 400 °C with 10 °C min^−1^ for 30 min (NiCe‐600H_2_ and NiCe‐750H_2_) while reaction was performed under 7 mbar of CO_2_:H_2_ gas mixture with 1:4 molar ratio using similar heating protocol (NiCe‐600R and NiCe‐750R). After treatment the reaction chamber was evacuated and the sample transferred rapidly under vacuum to the analysis chamber for XPS characterization. Survey and high‐resolution Ce 3d, Ni 2p, C 1s, O 1s XPS were collected using 44 eV pass energy and 0.1 eV energy step. Au 4f spectra (signal from supporting gold foil) was also recorded for binding energy (BE) calibration purpose. Peak fitting of Ce 3d peaks was performed using the CasaXPS software (Casa Software Ltd., version 2.3.25) and peak profiles previously recorded on reference samples.

##### Synchrotron‐Based Characterization

Combined AP‐XPS, AP‐HAXPES, and near‐edge X‐ray absorption fine structure (NEXAFS) measurements were conducted at the CAT@EMIL beamline at the synchrotron radiation facility BESSY II of the Helmholtz Zentrum, Berlin. This beamline equipped with a SPECS PHOIBOS 150 NAP hemispherical energy analyzer with a 2D‐CMOS detector provided access to both soft and hard X‐ray radiations enabling depth profiling from the surface to the subsurface. The sample (10 mg of pelletized NiCeO_*x*_ powder) was heated using IR laser targeting the plate at the back side of the pellet, in contact with a thermocouple. The gas flows (H_2_, O_2_) were introduced into the sample chamber via calibrated mass flow controllers (Bronkhorst) while the gas phase composition was monitored with a differentially pumped quadrupole mass spectrometer (Pfeiffer PrismaPro) connected to the chamber via a leak valve. The sample was alternatively treated and examined under 1 mbar of O_2_ at 450, 600, and 750 °C, each time followed by reduction at 400 °C under 1 mbar of H_2_ for 30 min. Photoemission spectra of Ce 3d and Ni 2p were collected using two‐photon energies (1065 eV for soft X‐rays and 4900 eV for tender X‐rays), the probing depth being estimated to be near 1.8 and 16.2 nm, respectively. The BE scale was aligned to the Ce 3d_3/2_ high‐BE satellite peak at 916.8 eV. The peak areas were estimated after background subtraction using the CasaXPS software. Atomic percentages were calculated after normalization of the photoemission peaks to the photon flux and the total photoionization cross section. The Ce 3d photoemission spectra were fit using standard reference curves of Ce^3+^ and Ce^4+^ recorded at the same spectrometer. The Ni L_3,2_‐edge NEXAFS spectra were obtained by collecting total electron yield (TEY) signal.

In‐situ X‐ray absorption spectra (XAS) were collected at the BM23 beamline of the European Synchrotron Radiation Facility.^[^ ^62^ ^]^ The samples were pressed in the form of self‐supporting pellets of 13 mm diameter and ≈50 mg of mass and placed in the Microtomo reactor cell suitable for thermal treatments under controlled gas atmosphere.^[^ ^63^ ^]^ Three ionization chambers were employed to measure incoming beam intensity (*I*_0_), transmitted beam (*I*_1_), and beam transmitted by a reference Ni metal foil (*I*_2_) placed after *I*_1_. XAS spectra of the samples were collected in fluorescence mode with Vortex Si detector. In situ XAS experiments were performed using the Microtomo reactor cell following the protocol reported in Figure S4 (Supporting Information). Pure He, H_2_, and CO_2_ were used as reactants. The total flow was kept constant at 50 mL min^−1^ while He:H_2_ 1:1 and CO_2_:H_2_ 1:4 ratios were employed during activation and reaction, respectively. Ni K‐edge spectra of the steady states were obtained as the average of five consecutive scans collected in step‐scan mode in the energy range 8180–9330 eV with 2 s/point integration time and energy resolution of 5 eV/point in the pre‐edge, 0.3 eV/ point in the XANES, and 0.035 Å^−1^/point in the EXAFS regions. NiO reference spectrum was measured on a 13 mm pellet in transmission mode. Given the lack of available spectra showing exclusively square planar Ni coordination within CeO_2_, the previously reported XAS spectrum for reduced NiCeO_2_,^[^ ^27^ ^]^ which exhibits characteristics of square planar Ni, is employed as a reference spectrum, henceforth referred to as Ni^δ+^ for brevity. Spectra energy calibration and alignment, background subtraction, edge jump normalization, EXAFS extraction, Fourier‐transform (FT) calculation, and linear combination fit (LCF) were performed with a Larch‐based python code.^[^ ^64^ ^]^ Relative abundance of Ni^δ+^, NiO, and Ni was evaluated through fit (*f*_*i*_) of each *i* ^th^ XANES spectrum with the LCF reported in the equation between the spectra of reference Ni^δ+^ (S_Niδ+_), NiO (S_NiO_), and Ni(S_Ni_).
(3)

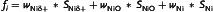




EXAFS fit was performed in the 8320–8430 eV energy range with the Artemis software from the Demeter package.^[^ ^65^ ^]^ The fit coefficients (w_Niδ+_, w_NiO_, and w_Ni_) were constrained to be positive and sum to unity. The goodness of fit was assessed using the R‐factor, as shown in Figure S3 (Supporting Information). Spectra representative of the steady state were obtained as the average of five scans measured in step‐scan mode. FT‐EXAFS spectra were extracted in the 2.3–12.6 Å^−1^ k‐range while the fit was performed in the 1–3.1 Å R‐range. Cubic NiO and cubic Ni were used as input structures in FEFF 6.0 to calculate Ni–O_NiO_, Ni–Ni_NiO_, and Ni–Ni_Ni_ scattering path (the subscript indicates the corresponding crystallographic phase). Amplitude reduction factor (S_0_^2^) was evaluated from fit of reference NiO reported in SI (Figure S10 and Table S4, Supporting Information).

## Conflict of Interest

The authors declare no conflict of interest.

## Author Contributions

**Mathias Barreau**: conceptualization (lead); data curation (equal); formal analysis (equal); investigation (lead); methodology (equal); validation (equal); writing—original draft (equal). **Davide Salusso**: data curation (equal); formal analysis (equal); resources (supporting); writing—original draft (supporting). **Jinming Zhang**: investigation (supporting). **Anna Efimenkoe**: resources (supporting). **Elisa Borfecchia**: validation (supporting). **Kamil Sobczak**: formal analysis (supporting); investigation (supporting). **Spyridon Zafeiratos**: conceptualization (lead); data curation (equal); formal analysis (equal); funding acquisition (lead); investigation (equal); methodology (equal); project administration (lead); resources (lead); supervision (lead); validation (lead); visualization (equal); writing—original draft (equal).

## Supporting information

Supplementary Material

## Data Availability

The data that support the findings of this study are available from the corresponding author upon reasonable request.
