# Supplementary material for: Thermal Activation and Deactivation of Ni‐Doped Ceria Catalysts in CO2 Methanation
Source: Small Sci. 2025 Feb 6;5(5):2400540. doi: 10.1002/smsc.202400540 (PMC12087771; doi:10.1002/smsc.202400540)
Supplement: Supplementary file 1 — Supplementary Material [file SMSC-5-2400540-s001.pdf]

Supporting Information

Thermal Activation and Deactivation of Ni-doped Ceria Catalysts in CO_2 Methanation

Mathias Barreau[a,§,*], Davide Salusso[b], Jinming Zhang[a], Michael Haevecker[c,d], Detre Teschner[c,d], Anna Efimenko[e,f], Elisa Borfecchia[g], Kamil Sobczak[h], and Spyridon Zafeiratos[a,*]

[a]*Institut de Chimie et Procédés pour l'Energie, l'Environnement et la Santé (ICPEES), ECPM, UMR 7515 CNRS – Université de Strasbourg, 25 rue Becquerel, 67087 Strasbourg Cedex 02, France*

[b]*European Synchrotron Radiation Facility, CS 40220, Cedex 9 F-38043 Grenoble, France*

[c]*Max-Planck-Institut für Chemische Energiekonversion (MPI-CEC), Stiftstrasse 34-36, D-45470 Mülheim a.d. Ruhr, Germany*

[d]*Fritz-Haber-Institut der Max-Planck-Gesellschaft, Faradayweg 4-6, D-14195 Berlin, Germany*

[e]*Interface Design, Helmholtz-Zentrum Berlin für Materialien und Energie GmbH (HZB), Albert-Einstein-Str. 15, 12489 Berlin, Germany*

[f]*Energy Materials In-situ Laboratory Berlin (EMIL), Helmholtz-Zentrum Berlin für Materialien und Energie GmbH (HZB), Albert-Einstein-Str. 15, 12489 Berlin, Germany*

[g]*Department of Chemistry, INSTM Reference Center and NIS Centers, University of Torino, 10125 Torino, Italy*

[h]*Faculty of Chemistry, Biological and Chemical Research Centre, University of Warsaw, Zwirki, Wigury 101, 02-089 Warsaw, Poland*

*mathias.barreau@ensicaen.fr *spiros.zafeiratos@unistra.fr

[§]Present address: M. Barreau, University of Caen Normandy

X-ray diffraction patterns

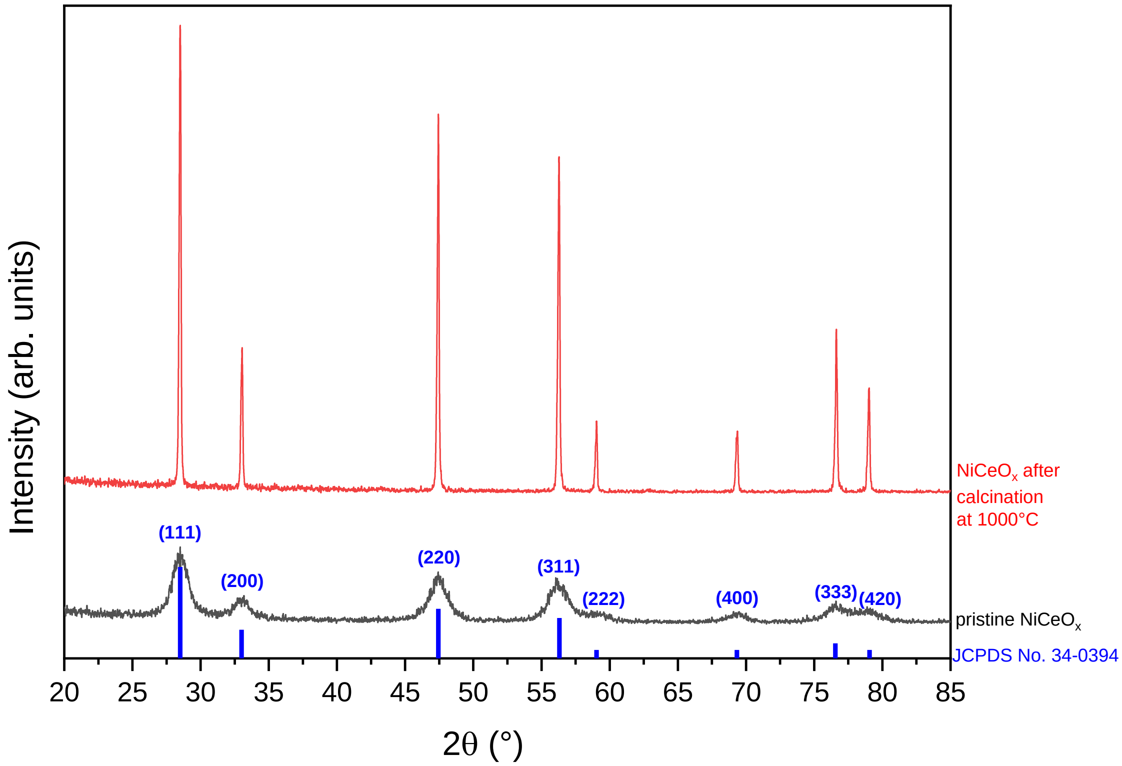

Figure S 1. XRD patterns of the $NiCeO_x$ catalyst before annealing (pristine) and after annealing at 1000 °C in synthetic air flow, both recorded at room temperature. The reference pattern for face-centered cubic cerium oxide (JCPDS card no. 34-394) is shown at the bottom for comparison.

AP-HAXPES spectra

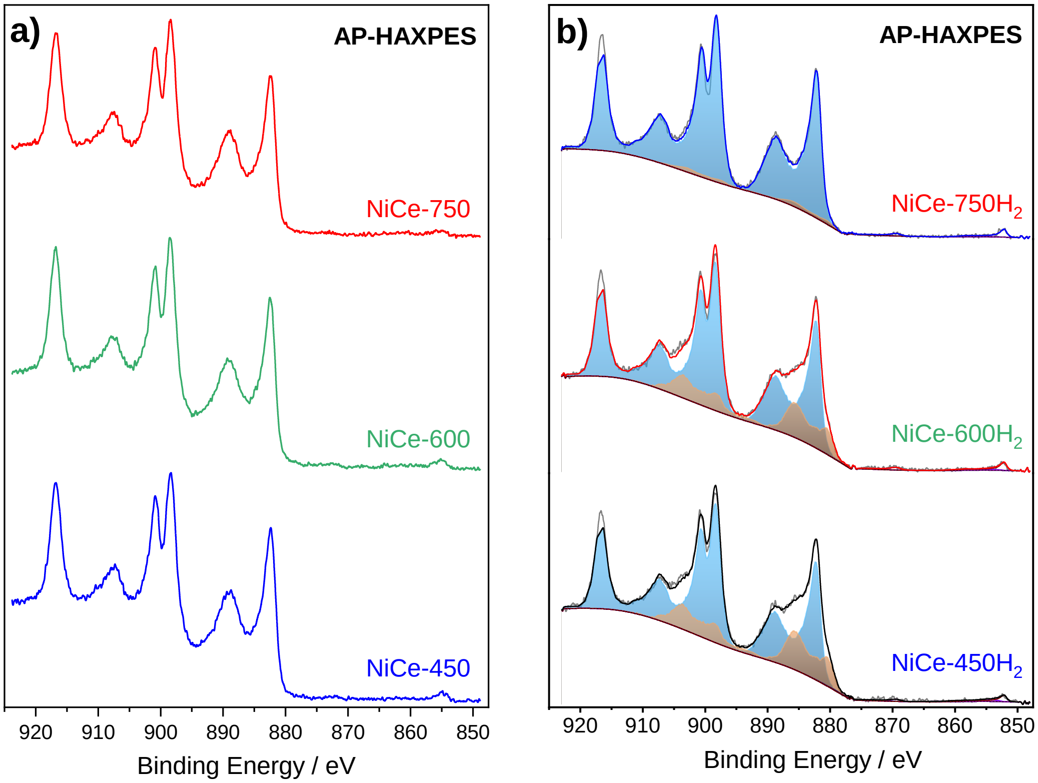

Figure S2. AP-HAXPES Ni 2p and Ce 3d spectra of **(a)** NiCe-450, NiCe-600 and NiCe-750 measured at 400 °C in 1 mbar O_2 and **(b)** NiCe-450H_2, NiCe-600H_2 and NiCe-750H_2 measured at 400 °C in 1 mbar H_2. Depth of the photoemission measurements is estimated to be 16.2 nm.

Table S 1 .The %Ni at various calcination temperatures as calculated based on the AP-XPS and AP-HAXPES results shown in Figure 3c of the manuscript. The % excess Ni at the surfaces is calculated as % [(Ni(AP-XPS)-Ni(AP-HAXPES))/ Ni(AP-HAXPES)].

Temp (°C)	%Ni (AP-XPS)	%Ni (AP-HAXPES)	Surface Ni excess (%)
450	8.0	4.7	70
600	15.0	5.6	170
750	7.9	4.4	80

In situ X-Ray based synchrotron characterization (XANES)

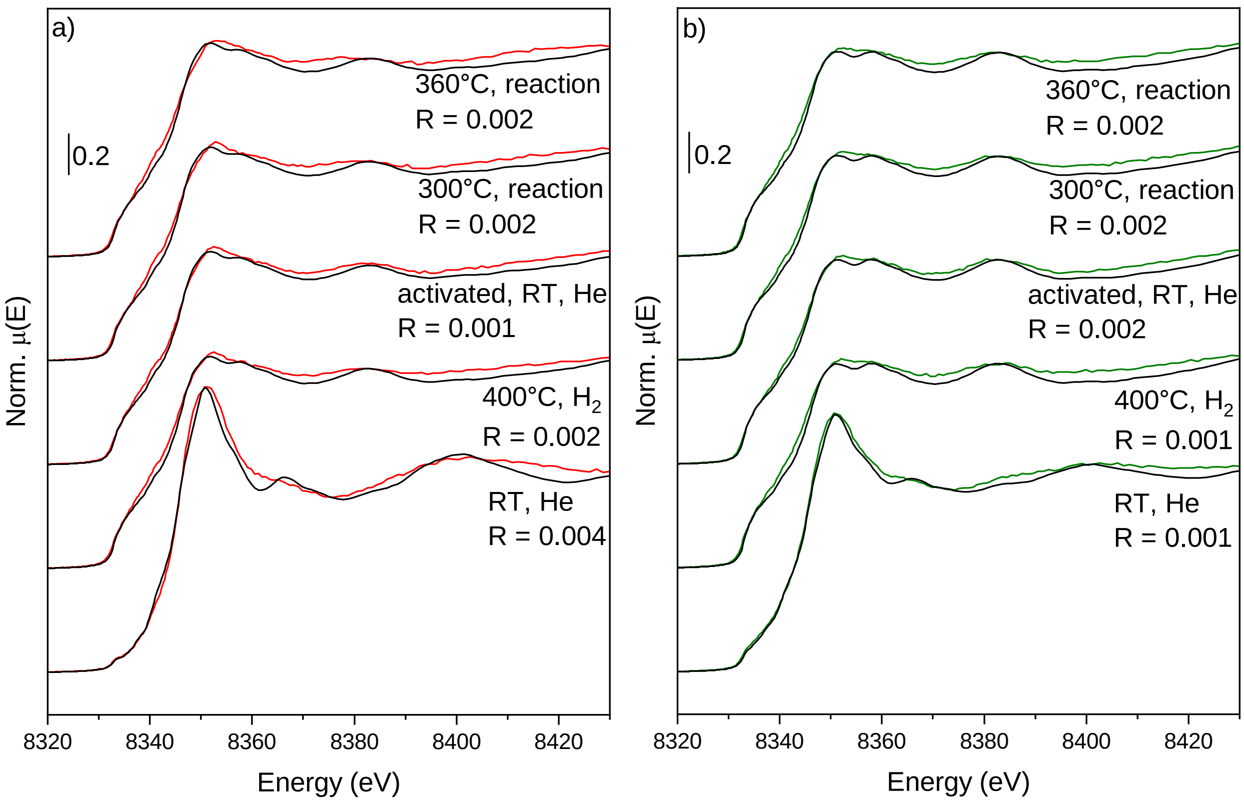

Figure S3. Experimental spectra (colored line) and best fit curve (black line) for representative states of **(a)** NiCe-600 (red line) and **(b)** NiCe-750 (green line) taken from steady states in protocol represented in Figure S4.

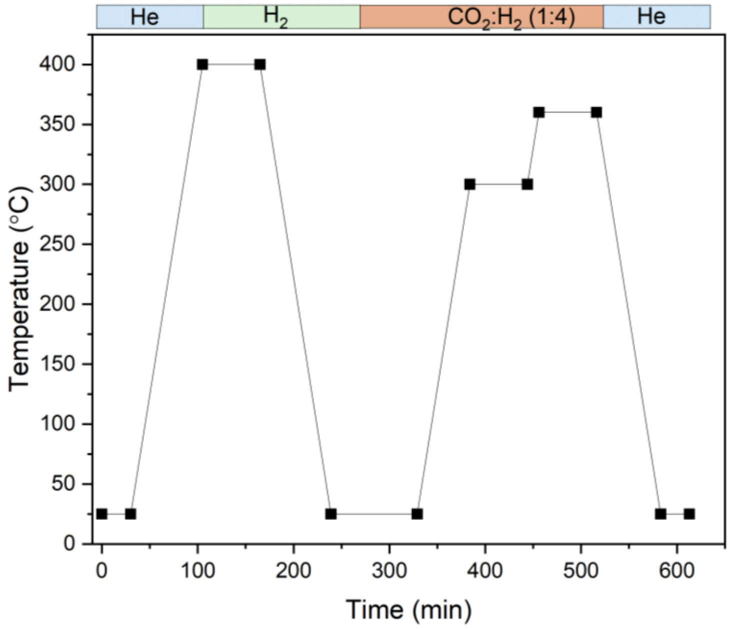

Figure S4. Scheme of the reaction protocol employed for in-situ XAS measurements. The total flow was kept constant at 50 mL min^{-1}.

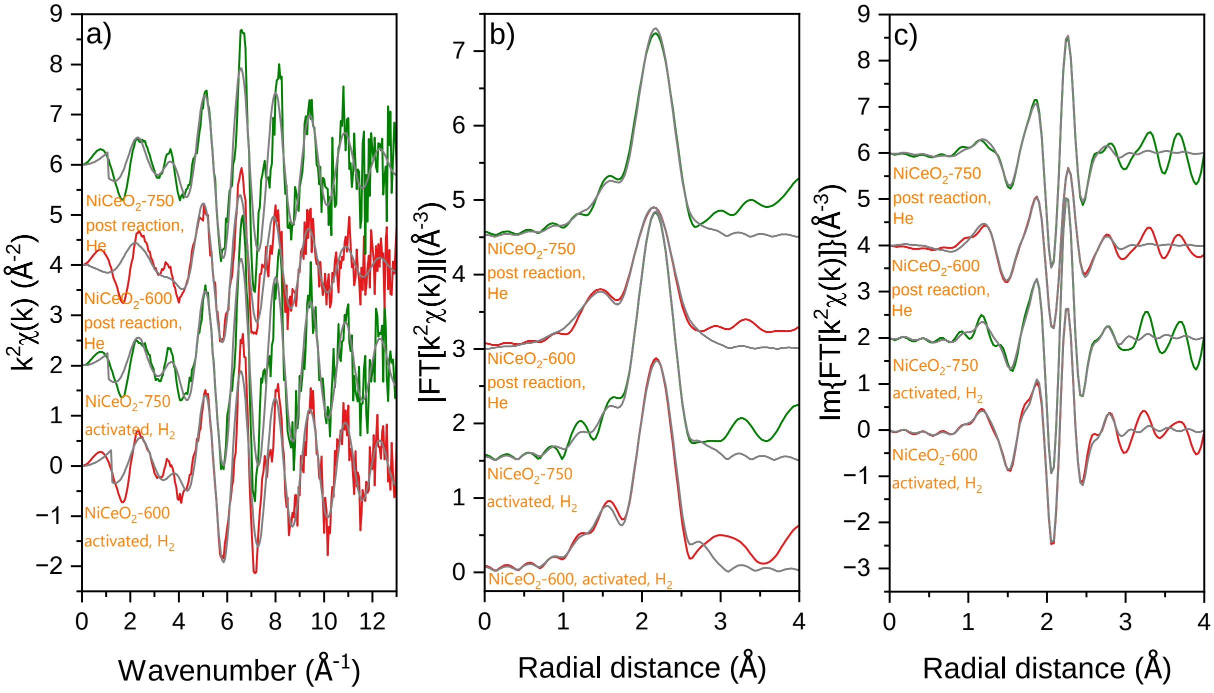

Figure S 5 In-situ Ni K-edge experimental spectra of $NiCeO_2$-600 (red line) and $NiCeO_2$-750 (green line) and best fit curves (gray line) of **(a)** $k^2(k)$ EXAFS functions and FT-EXAFS **(b)** magnitude and **(c)** imaginary components. Spectra represent the state of the catalysts at RT under H_2 gas flow before reaction (star symbol) and at RT under He gas flow after reaction (left pointing triangle symbol).

Table S 2 FT-EXAFS fit results of spectra reported in Figure S5 S_0^2 was fixed at 0.92 as evaluated from fit of NiO. CN were weighted for the phases respective abundances extracted from LCF analysis.

	$NiCeO_2$-600-activated (H_2)	$NiCeO_2$-750-activated (H_2)	$NiCeO_2$-600-post reaction (He)	$NiCeO_2$-750-post reaction (He)
R-factor	0.02	0.011	0.014	0.011
$N_{var}(N_{ind})$	9(14)	9(14)	9(14)	9(14)
ΔE_0 (eV)	5 ± 2	5 ± 2	1 ± 2	4 ± 2
CN $(Ni\text{-}Ni)_{Ni}$	7.1 ± 1.3	8.0 ± 1.1	7 ± 1	8.6 ± 1.4
R $(Ni\text{-}Ni)_{Ni}$ (Å)	2.474 ± 0.011	2.468 ± 0.008	2.464 ± 0.014	2.471 ± 0.009
σ^2 $(Ni\text{-}Ni)_{Ni}$ (Å^2)*	0.0045 ± 0.0013	0.0054 ± 0.0009	0.008 ± 0.0016	0.0073 ± 0.0012
CN $(Ni\text{-}O)_{NiO}$	3 ± 2	4 ± 2	4 ± 1	3 ± 2
R $(Ni\text{-}O)_{NiO}$(Å)	1.86 ± 0.04	1.89 ± 0.05	1.82 ± 0.02	1.88 ± 0.03
σ^2 $(Ni\text{-}O)_{NiO}$ (Å^2)	0.004 ± 0.008	0.009 ± 0.002	0.004 ± 0.003	0.002 ± 0.002
CN$(Ni\text{-}Ni)_{NiO}$	9 ± 2	8 ± 2	8 ± 1	9 ±2
R $(Ni\text{-}Ni)_{NiO}$ (Å)	2.98 ± 0.06	2.94 ± 0.07	2.9 ± 0.04	2.91 ± 0.08
σ^2 $(Ni\text{-}Ni)_{NiO}$ (Å^2)*	0.014 ± 0.007	0.014 ± 0.010	0.013 ± 0.005	0.018 ± 0.009

* indicates parameters that were constrained to the same value for both the metallic and the oxidized Ni-phases.

STEM/EDX Characterization of the spent NiCe-600R and NiCe-750 catalysts

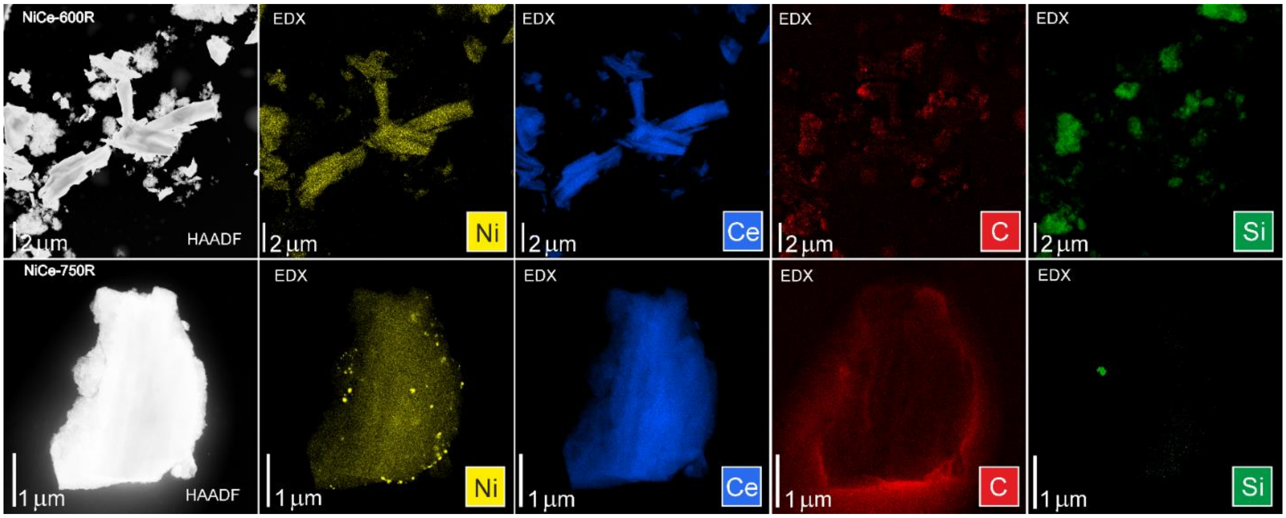

Figure S6. Low magnification TEM and TEM/EDX mapping images of NiCe-600R (top) and NiCe-750R (bottom) catalysts. The Si signal corresponds to the SiC used to load the sample in the catalytic reactor.

Raman Characterization

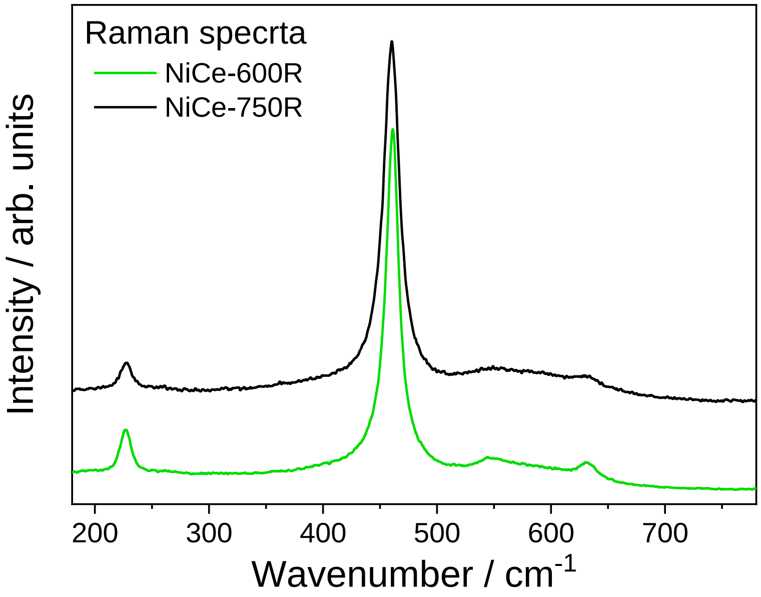

Figure S7. Raman spectra of NiCe-600R and NiCe-750R catalysts recorded with a 532 nm laser.

STEM/EDX concentration line profiles

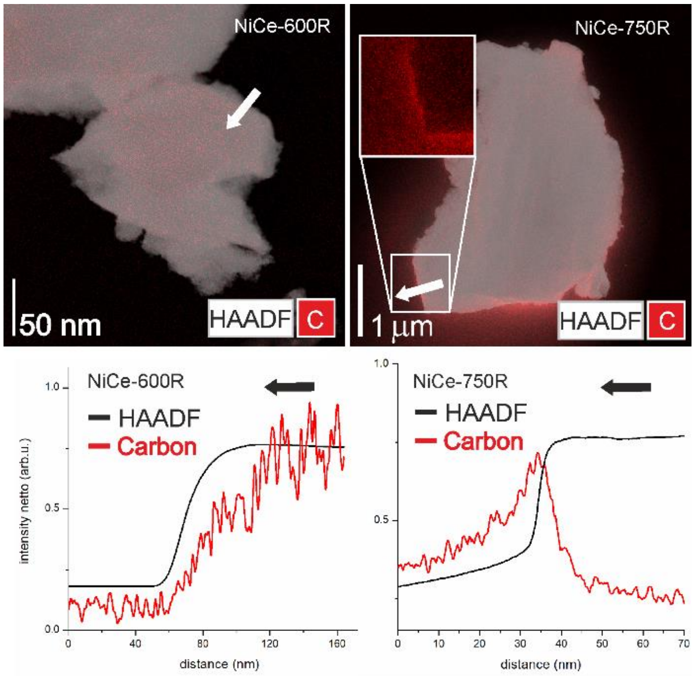

Figure S8. Characteristic line-scanning EDX of C element at the edge of NiCe-600R (left) and NiCe-750R (right) spent catalysts.

Quasi In situ XPS

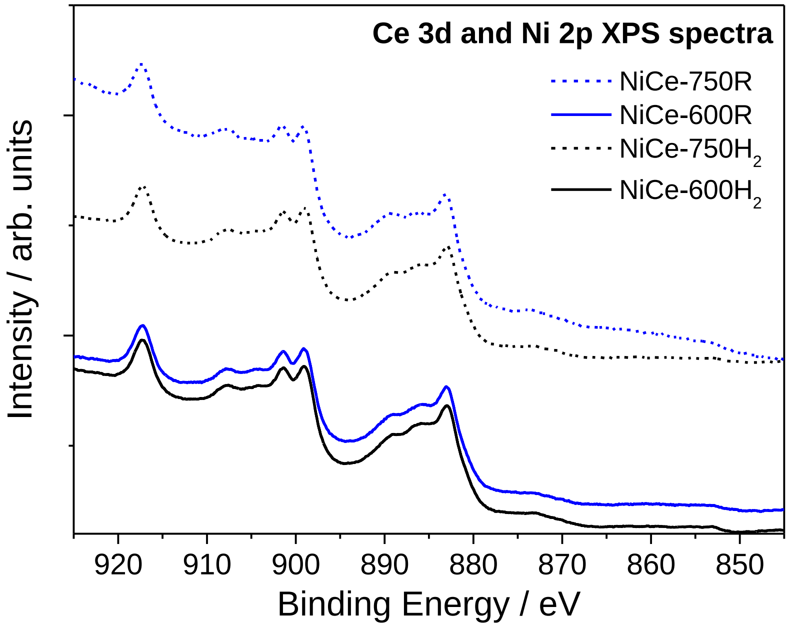

Figure S9. Quasi in situ XPS Ce 3d and Ni 2p spectra of NiCe-600R (blue solid line) and NiCe-750R (blue dashed line) of the spent catalysts. The corresponding spectra recorded just before the reaction (i.e. NiCe-600H_2 and NiCe-750 H_2) are included for comparison. Experimental conditions: LO test up to 400 °C with 10 °C.min^{-1} heating rate followed by 30 min step at 400 °C, under 7 mbar of CO_2:H_2 gas mixture with 1:4 molar ratio. Sample previously reduced in 7 mbar H_2 following similar heating procedure.

Table S3. Ce 3d/ Ni 2$p_{3/2}$ atomic ratios estimated from quasi in situ measurements presented in Figure S9.

Sample	Precise conditions	Calcination Temperature / °C	
		600	750
NiCe-xxxH_2	7 mbar H_2, 400°C	4,7	5,1
NiCe-xxxR	7 mbar CO_2:H_2 (1:4), 400°C	4,5	3,0

XANES Analysis: Example of NiO Spectrum

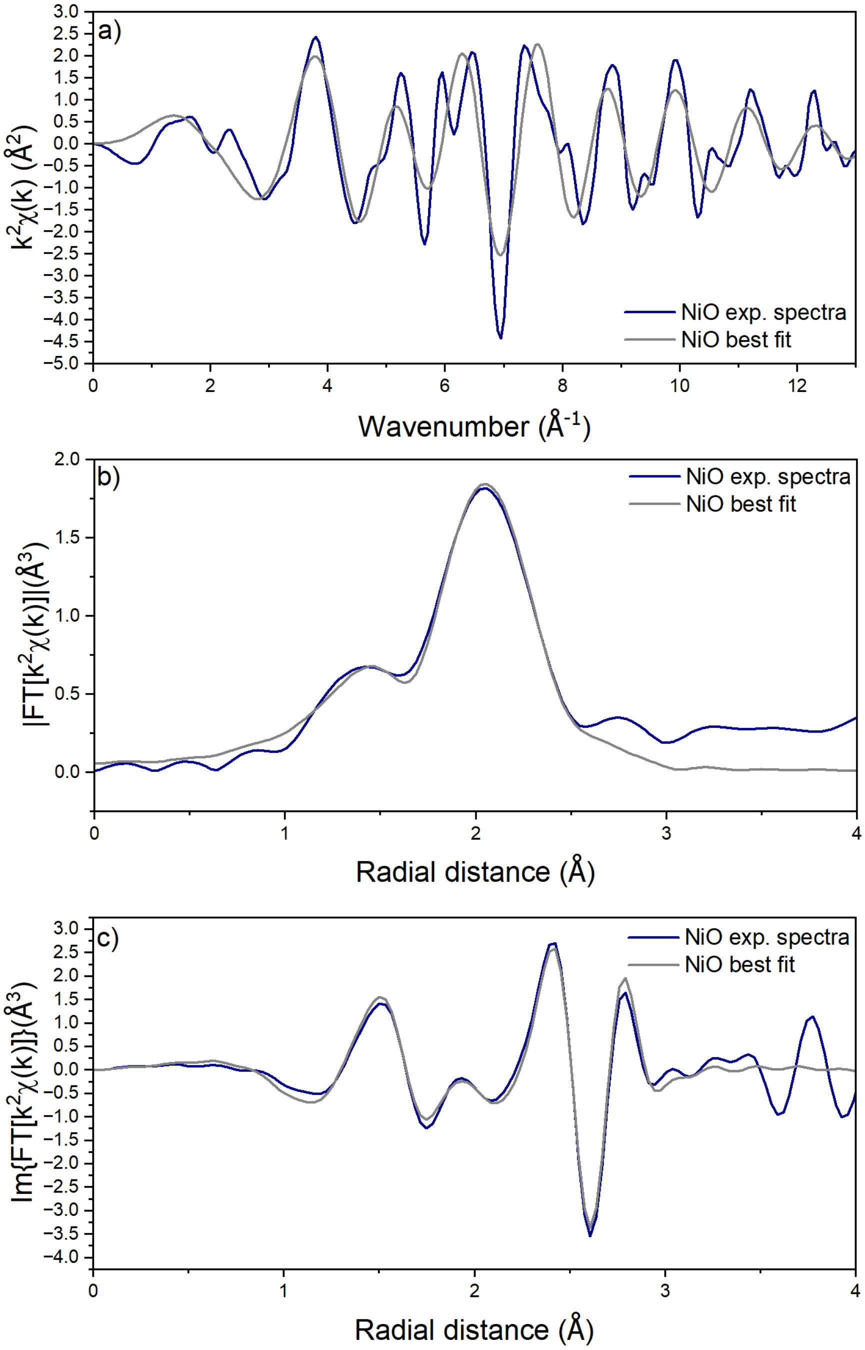

Figure S 10 Reference NiO Ni K-edge experimental spectra (blue line) and best fit curve (gray line) of **(a)** EXAFS and FT-EXAFS **(b)** magnitude and **(c)** imaginary components.

Table S 4 FT-EXAFS fit results of reference NiO. FT-EXAFS spectrum was extracted in the 2.5 – 13.9 Å^{-1} k-range while fit was performed in the 1 – 3 Å R-range.

	NiO
R-factor	0.016
N_{var}(N_{ind})	6(14)
ΔE_0 (eV)	-3 ± 1
S_0^2	0.92 ± 0.09
CN (Ni-O)$_{NiO}$	6*
R (Ni-O)$_{NiO}$ (Å)	2.071 ± 0.012
σ^2 (Ni-O)$_{NiO}$ (Å^2)	0.0072 ± 0.0018
CN (Ni-Ni)$_{NiO}$	12*
R (Ni-Ni)$_{NiO}$ (Å)	2.956 ± 0.007
σ^2 (Ni-Ni)$_{NiO}$ (Å^2)	0.0065 ± 0.0007

* indicates parameters that were constrained to crystallographic values.
